# A scoping review to create a framework for the steps in developing condition-specific preference-based instruments de novo or from an existing non-preference-based instrument: use of item response theory or Rasch analysis

**DOI:** 10.1186/s12955-024-02253-y

**Published:** 2024-05-14

**Authors:** Teresa C. O. Tsui, Sofia C. Torres, Joanna M. Bielecki, Nicholas Mitsakakis, Maureen E. Trudeau, Karen E. Bremner, Aileen M. Davis, Murray D. Krahn

**Affiliations:** 1 Toronto Health Economics and Technology Assessment (THETA) Collaborative, University Health Network, Toronto, ON Canada; 2 Canadian Centre for Applied Research in Cancer Control, Toronto, ON Canada; 3 Child Health and Evaluative Sciences, Hospital for Sick Children, Toronto, ON Canada; 4 Institute of Health Policy, Management and Evaluation, Dalla Lana School of Public Health, University of Toronto, Toronto, ON Canada; 5 Centro Hospitalar Universitário Lisboa Norte, Lisboa, Portugal; 6 Children’s Hospital of Eastern Ontario, Ottawa, ON Canada; 7 Odette Cancer Centre, Sunnybrook Health Sciences Centre, Toronto, ON Canada

**Keywords:** Scoping review, Health-related quality of life, Condition-specific preference-based instrument, Framework, Rasch analysis, Item response theory

## Abstract

**Background:**

There is no widely accepted framework to guide the development of condition-specific preference-based instruments (CSPBIs) that includes both de novo and from existing non-preference-based instruments. The purpose of this study was to address this gap by reviewing the published literature on CSPBIs, with particular attention to the application of item response theory (IRT) and Rasch analysis in their development.

**Methods:**

A scoping review of the literature covering the concepts of all phases of CSPBI development and evaluation was performed from MEDLINE, Embase, PsychInfo, CINAHL, and the Cochrane Library, from inception to December 30, 2022.

**Results:**

The titles and abstracts of 1,967 unique references were reviewed. After retrieving and reviewing 154 full-text articles, data were extracted from 109 articles, representing 41 CSPBIs covering 21 diseases or conditions. The development of CSPBIs was conceptualized as a 15-step framework, covering four phases: 1) develop initial questionnaire items (when no suitable non-preference-based instrument exists), 2) establish the dimensional structure, 3) reduce items per dimension, 4) value and model health state utilities. Thirty-nine instruments used a type of Rasch model and two instruments used IRT models in phase 3.

**Conclusion:**

We present an expanded framework that outlines the development of CSPBIs, both from existing non-preference-based instruments and de novo when no suitable non-preference-based instrument exists, using IRT and Rasch analysis. For items that fit the Rasch model, developers selected one item per dimension and explored item response level reduction. This framework will guide researchers who are developing or assessing CSPBIs.

**Supplementary Information:**

The online version contains supplementary material available at 10.1186/s12955-024-02253-y.

## Plain language summary

Preference-based instruments are questionnaires that generate “utility”, a global measure of quality of life anchored at 0 for “dead” to 1 for “full health”. Condition-specific preference-based instruments assess utility on dimensions that are relevant to individuals living with specific health problems. We reviewed the published literature on condition-specific preference-based instruments to develop a framework of steps to guide and standardize their development. Our focus was the use of Rasch analysis and item response theory, statistical methods to select the core questions. Using these methods, the resulting instruments are more precise as fewer questions can represent the key dimensions.

We found 109 articles which described the development of 41 condition-specific preference-based instruments for 21 different diseases or conditions. The development of condition-specific preference-based instruments was conceptualized as a 15-step framework, covering four phases: 1) develop initial questionnaire items, 2) establish the dimensions of the items, 3) reduce the number of items per dimension, and 4) value and model health state utilities. These phases extend from the development of the non-preference-based instrument and multi-attribute classification system to the preference-based instrument. This framework will improve the development of instruments to measure utilities for specific conditions, which will enable the use of high-quality utilities in clinical decisions and cost-effectiveness analyses.

## Introduction

Condition-specific preference-based instruments (CSPBI) measure health-related quality of life (HRQoL) relevant to patients with a specific condition or disease. In contrast, generic preference-based instruments such as the EQ-5D family of questionnaires [1] are suitable for general use [1–3]. Preference-based instruments contain a classification system with items representing attributes and levels within items which, with a value set, produce a utility score anchored at zero (dead) and one (perfect health). Values are derived from patients or members of the general public who provided utilities for health states using direct methods, including time trade off (TTO) [3, 4] or discrete choice experiments (DCE) [3, 4]. Utility is used to calculate the quality-adjusted life year (QALY), a key outcome in economic evaluations and clinical decision-making.

Guidelines recognize that CSPBI discriminate between known groups better than generic instruments and are more responsive to changes in disease-specific dimensions [5–9].

Several systematic reviews included aspects of CSPBI development, including one that found 51 different CSPBIs [5, 10–13]. Brazier et al. [5] described six stages of preference-based instrument development starting with an existing condition-specific non-preference-based instrument, such as the Functional Assessment of Cancer Therapy – General measure (FACT-G) scale [14], or European Organization for Research and Treatment of Cancer (QLQ-C30) [15] in oncology. The stages are: I) establish dimensionality, II) select items for each dimension, III) test the number of levels, IV) validate the health state classification system, V) valuation survey, and VI) model the valuation data. When there is no established condition-specific non-preference-based instrument, the steps in the development of a CSPBI begin with creating a classification system of domains de novo [13, 16].

Factor analysis (confirmatory or exploratory) is used to establish dimensions. Item response theory (IRT) or Rasch analysis can be used to eliminate items and select one or two items to represent each dimension [5]. Item response theory (IRT) is a measurement approach that explains the probabilistic relationship between items and a latent construct (e.g., HRQoL) [17]. The Rasch model is the simplest IRT model [18]. When items fit the Rasch model, the instrument has favourable properties: unidimensionality, interval-level scoring, additivity, and sample-free measurement [19]. Instruments developed with Rasch or IRT methods have high precision and efficiency by selecting the fewest items to cover the latent construct [19, 20]. Health states are then sampled and modelled using a decomposed or composite approach [5]. While these stages provide a starting point for the development of novel CSPBI, the methods described by Brazier et al. begin with an existing condition-specific non-preference-based instrument. Within these stages, there are insufficient details for novice CSPBI developers to follow. Additionally, when there is no suitable condition-specific non-preference based instrument, developing a novel CSPBI de novo is the best option. These initial steps of creating a non-preference-based instrument de novo have been described by Guyatt et al. [16], yet these steps were absent from the Brazier et al. stages [5].

The aim for this scoping review is to address these gaps by operationalizing Brazier et al.’s stages based on available literature, and adding the initial steps to develop a preference-based instrument de novo when there is no existing HRQoL instrument. Our focus was the use of Rasch and IRT methods to establish dimensions, reduce items per dimension, and reduce item levels because resulting instruments have favourable properties. These steps underpin the creation of a multi-attribute health state classification system to develop a novel preference-based instrument. Our objectives were to:

1. Identify the steps in constructing CSPBIs, both de novo and from an existing non-preference-based instrument.
2. Describe the application of Rasch or IRT methods within these steps.
3. Develop an expanded framework to guide future development of CSPBIs.

## Methods

### Information sources

We followed the Joanna Briggs Institute (JBI) published guidance document [21, 22], and the Preferred Reporting Items for Systematic Review and Meta-Analysis Scoping Review (PRISMA-ScR) reporting guidelines (Supplementary Information, S1) [23]. Our scoping review protocol was not published.

Searches were performed in Ovid MEDLINE, Ovid Embase, Ovid PsychInfo, EBSCO CINAHL, and the Cochrane Library from inception to December 2022 (Supplementary Information, S2). An experienced health sciences librarian (JB) and TT developed a search strategy (Supplementary Information, S2) using Medical Subject Headings (MeSH) and keywords about:

1. Measurement of condition-specific HRQoL
2. Eliciting health state utility values to develop a preference-based instrument
3. Methods to develop instruments measuring HRQoL
4. IRT including Rasch analysis

The search strategy was reviewed by a second librarian, following the Peer Review of Electronic Search Strategies (PRESS) standard [24].

### Selection of articles

Search results were imported into Thomson Reuters EndNote X9.3.3 to remove duplicates.

A primary (TT) and secondary (ST) reviewer independently screened titles and abstracts, followed by full text articles using Covidence [25]. We excluded abstracts, commentaries, editorials, letters, and non-English articles. Articles were excluded if they predicted utilities from only demographics or other non-disease factors, or validated non-English instruments, since these do not describe the development of the instruments.

We included articles that described either the development of a CSPBI using IRT or Rasch analysis, or the elicitation of utility weights for the instrument. Articles about instruments had the following measurement purposes: 1) to discriminate between known disease states, or 2) to measure responsiveness after treatment and over time.

We also hand-searched the reference list of Goodwin’s systematic review [13] for the names of instruments. Additional searches were performed using individual instrument names on Pubmed from inception to February 2024 (Supplementary Information, S3). We chose the review by Goodwin and Green because it included all steps of CSPBI development, and was the most recent and most comprehensive of the review papers that we found.

Inter-reviewer reliability was assessed using a kappa statistic, with cut-off scores: 0.40–0.59 for *fair* agreement, 0.60–0.74 for *good* agreement, and 0.75 and higher for *excellent* agreement [26]. Discrepancies in interpreting eligibility criteria were discussed, and the criteria were revised for clarity if inter-reviewer reliability was below good [26].

### Data extraction

The steps of instrument development were extracted from full text articles. The data extraction form (Supplementary Information S4) was pilot tested on 10 articles that covered all instrument development phases and was iteratively revised until it captured all essential steps. One reviewer (TT) extracted the data from all articles and a second reviewer (ST) reviewed the data against all articles. Discrepancies were resolved by discussion.

### Constructing the framework

We started with Brazier et al.’s six stages outlining how to derive CSPBIs from existing psychometric instruments [5]. Next, we reviewed existing frameworks for the development of classification systems of domains for non-preference-based instruments [16, 27], and for use of factor analysis [28, 29] and Rasch analysis [19]. Finally, we reviewed articles describing the development of CSPBIs to identify the key steps.

## Results

### Study selection

**Fig. 1:**
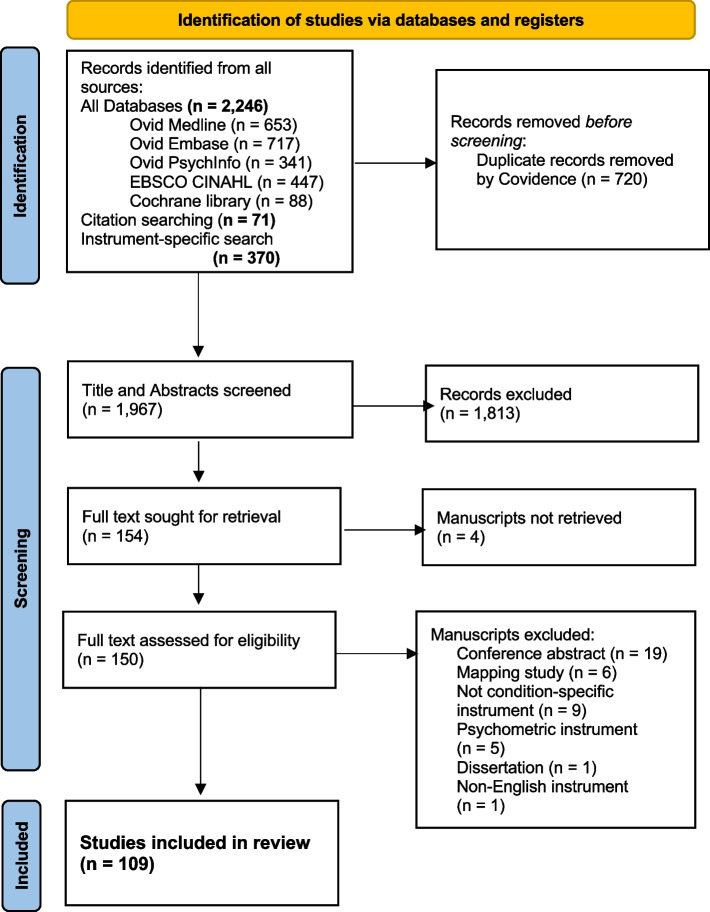
PRISMA Diagram [30]

Figure 1 shows the PRISMA diagram. After removal of duplicates, the titles and abstracts of 1,967 references were reviewed, and 71 additional references were identified from hand-searching Goodwin’s systematic review [13]. One hundred and fifty-four full-text articles were retrieved and reviewed. Data were extracted from 109 articles representing 41 unique instruments, and 21 diseases/conditions. Inter-rater agreement was fair (kappa = 0.57) during the title and abstract screening, and good (kappa = 0.71) during the full text selection.

### Study characteristics

Table 1 outlines the 41 CSPBIs, which covered 21 diseases or conditions. Instruments had 4 to 10 dimensions (median = 6). Only the classification systems were developed for three instruments, without preference elicitation. Direct preference elicitation methods were used for 38 instruments: TTO (22), TTO and rating scale (RS) (1), DCE (1), DCE with TTO (DCE_-TTO_) (5), DCE and DCE_-TTO_ (1), DCE_-TTO_ and best worst scaling (2), standard gamble (SG) (1), SG and RS (1), SG and VAS (1), and VAS alone (1). Sources of utilities (number of studies) were the general public only (22), patients only (7), general public and patients (7), general public and patient and care-giver dyads (1), and general public and carers (1) (Table 1).

**Table 1:** Published condition-specific preference-based instruments

Condition	Instrument [references]	Dimensions	Number of health states	Method of eliciting utilities	Groups for utility elicitation	Scaling anchors	Country
Aberrant behaviour	Aberrant Behavior Checklist -Utility Index (ABC-UI) [31, 32]	7	3^(7) = 2,187	TTO (LT-TTO worse than dead)	UK general public	Best(1), worst (0)	UK
Asthma	Asthma Quality of Life Questionnaire- 5 Dimensions (AQL-5D) [33–39]	5	5^(5) = 3,125	TTO	UK general public	Best (1), worst (0)	UK
Bladder problems	Incontinence Utility Index (IUI) [40, 41]	5	3^(5) = 243	TTO	UK general public	Perfect health (1), dead (0)	UK Spain
Bladder problems	Overactive Bladder Questionnaire- 5 Dimensions (OAB-5D) [9, 36, 42–44]	5	5^(5) = 3,125	TTO	UK general public	Full health (1), dead (0)	UK USA
Cancer	European Organisation for Research and Treatment in Cancer Core Quality of Life Questionnaire – 8 Dimensions (EORTC-8D) [7, 45–48]	8	81,920	TTO	UK general public Sri Lankan general public	Best (1), worst (0)	UK Sri Lanka
Cancer	European Organisation for Research and Treatment in Cancer Core Quality of Life Questionnaire – 10 Dimensions QLU-C10D [49–60]	10	4^(10) = 1,048,576	DCE-_TTO_ (Australia, France, Austria, Italy, and Poland) DCE (Canada, Netherlands, Germany, UK, U.S.)	General public	Best (1), worst (0)	Australia Netherlands Canada France Germany UK USA Austria, Italy, and Poland
Cancer	European Organisation for Research and Treatment in Cancer Core Quality of Life Questionnaire – Preference-Based Measure (QLQ-PBM) [61]	8	105	TTO	Dutch general public	Best (1),worst (-1) -1 to 0 worse than dead	Netherlands
Cancer	Functional Assessment of Cancer Therapy – 8 Dimensions (FACT-8D) [62, 63]	8	1744 participants; 16 choice pairs; 256 observations per choice set	DCE	Patients with common cancers	Best (1), worst (0)	Australia
Cancer (breast)	Breast Utility Instrument (BUI) [64, 65]	10	N/A	N/A	N/A No. utilities were elicited yet, so patients with breast cancer is an error.	N/A	Canada
Cerebral palsy	Cerebral Palsy-6 Dimensions (CP-6D) [66–68]	6	5^(6) = 15,625	DCE-_TTO_	Australian general public	Best (1), worst (0)	Australia
Cystic fibrosis	Cystic Fibrosis Questionnaire-Revised-8 Dimensions (CFQ-R-8 D) [69]	8	3^(8) = 6,561	TTO	UK general public	Best (1), worse than dead (< 0)	UK
Dementia	Dementia Quality of Life-Utility (patient self-report and carer proxy-report) DEMQOL-U DEMQOL-Proxy-U [8, 70–74]	5 4	4^(5) = 1,024 4^(4) = 256	TTO	UK general public and patients UK general public and carers	Best (1), worst (0)	UK
Dementia	Alzheimer’s Disease-5 Dimensions (AD-5D) [75–77]	5	4^(5) = 1,024	DCE_-TTO_ BWS	Australian general public and patients with dementia & carer dyads	Best (1), worst (0)	Australia
Diabetes	Diabetes Utility Index (DUI) [78, 79]	5	4^(5) = 1024	VAS SG	People with type 1 or 2 diabetes	Best (1), worst (0)	USA
Diabetes	Diabetes Health Profile-3 Dimensions and 5-Dimensions (DHP-3D and DHP-5D) [80]	3 5	33 were valued	TTO	UK general public	Best (1), worst (0)	UK
Diabetes	Health and Self-Management in Diabetes Questionnaire (HASMID) (HASMID-8 HASMID-10) [81, 82]	8 10	262,144 profiles 120 choice sets across 10 survey versions	DCE-_TTO_	UK general public and Individuals with diabetes	Unanchored and WTP estimates	UK
Duchenne Muscular Dystrophy	Duchenne Muscular Dystrophy Quality of Life-8 Dimensions (DMD-QoL-8D) [83]	8	4^(8) = 65,536	DCE-_TTO_	UK general public	Best (1), dead (0)	UK
Epilepsy	Epilepsy-specific Quality of Life – 6 Dimensions (NEWQOL-6D) [84–86]	6	4^(6) = 4,096	TTO	UK general public People with epilepsy	Best (1), worst (0)	UK
Heart disease	MacNew Heart Disease Health-Related Quality of Life Instrument -7 Dimensions (MacNew) [87]	7	4^(7) = 16,384	N/A	N/A	N/A	Australia
Human immunodeficiency virus (HIV)	Preference-based HIV index (PB-HIV)	7	3^(7) = 2,187	EQ-VAS	People with HIV	Best (100), and worst (0)	Canada
Mental health	Clinical Outcomes in Routine Evaluation-Outcome Measure – 6 Dimensions (CORE-6D) [88–90]	6	33 plausible health states (Rasch vignette approach) 3^(6) = 729 CORE-6D health states	TTO	UK general public	Full health (1), dead (0)	UK
Mental health	Recovering Quality of Life Utility Index (ReQoL-UI) [91–94]	7	66 health states valued Each participant valued 8 health states	TTO Lead-time TTO (states worse than dead)	Nationally representative sample from Scotland, England, Wales	Best (1), worst (0)	UK
Mobility	Mobility Quality of Life-7 Dimensions (MobQoL-7D) [95, 96]	7	4^(7)﻿ = 16,384	VAS	UK general public Representative sample of people with impaired mobility	Best (100), worst (0)	UK
Multiple sclerosis	Multiple Sclerosis Impact Scale – 8 Dimensions, and -8 Dimensions patient versions (MSIS-8D MSIS-8D-P) [27, 97–100]	8	4^(8)﻿ = 65,536	TTO	UK general public People with MS	Best (1), worst (0)	UK
Multiple sclerosis	Multiple Sclerosis Impact Scale – Preference-Based Measure (MSIS-PBM) [61]	6	100	TTO	Dutch general public	Best (1), worst (-1) -1 to 0 worse than dead	Netherlands
Multiple sclerosis	Health-related quality of life in people with neurological conditions (Neuro-QoL-Utility System (NQU)) [101, 102]	6	59 health states 47 single-attribute states 6 corner states 3 marker states: worst, best, dead	SG	General public sample, and patients with MS	Best (1), worst (0)	UK
Multiple sclerosis	Preference-Based Multiple Sclerosis Index (P-PBMSI) [103–106]	5	3^(5) = 243	SG RS	People with MS	Best (1), worst (0)	Canada
Myelofibrosis	Myelofibrosis-8 Dimensions (MF-8D) [107]	8	2560	TTO	UK general public	Best (1), worst (0)	UK
Obesity	Weight-specific Adolescent Instrument for Economic-Evaluation (WAITe) [108–110]	5	7^(5)﻿ = 16,807	TTO	UK general public	Best (1), worst (0)	UK
Obesity	Preference-Based Index of Weight-Related Quality of Life (PBI-WRQL) [111]	7	3^(7) = 2,187	EQ-VAS	Patients	Best (100), worst (0)	Canada
Oral health	Caries Impacts and Experiences Questionnaire for Children Utility version (CARIES-QC-U) [112, 113]	4	3^(5)﻿ = 243	DCE_-TTO_ BWS	Adolescents and adults from the general public	Best (1), worst (0)	UK
Oral health	Early Childhood Oral Health Impact Scale-4 Dimensions (ECOHIS-4D) [114, 115]	4	3^(4) = 81	DCE_-TTO_	Australian general public	Best (1), worst (0)	Australia
Palliative care	Palliative Care Outcome Scale Descriptive System (POS-E) [116]	3	3^(7) = 2187	N/A	N/A	N/A	UK
Rheumatoid arthritis	Multiattribute Health Outcome Measure for Rheumatoid Arthritis (MHOM RA) [117]	6	4^(6) = 40,096	TTO (general health preference)	Patients with rheumatoid arthritis	Best (1), worst (0)	UK
Rheumatoid arthritis	Health Assessment Questionnaire – Preference-Based Measure (HAQ-PBM) [61]	5	56	TTO	Dutch general public	Best (1), worst (0)	Netherlands
Vision	Vision Quality of Life Index (Vis-QoL) [118–124]	6	5^(6﻿) = 15,625	TTO and RS (intermediate item responses)	Patients with visual impairment (Canada) Patients with diabetic retinopathy, diabetic macular edema, and keratoconus (Australia) Patients with visual impairment (India)	Best (1), dead (0), States worse than dead -0.25	Canada Australia India
Vision	Visual Function Questionnaire – Utility Index (VFQ-UI) [125–130]	6	5^(6)﻿ = 15,625	TTO	General public from Australia, Canada, UK, USA	Best (1), worst (0)	Australia, Canada, UK, USA

*Abbreviations*: *TTO* Time trade-off, *DCE-TTO* Discrete-choice experiment, time-trade-off, *LT-TTO* Lead time-time trade-off, *BWS* Best worst scaling, *RS* Rating scale, *VAS* Visual analogue scale, *UK* United Kingdom, *USA* United States of America

### Phases and steps to developing CSPBIs

**Fig. 2:**
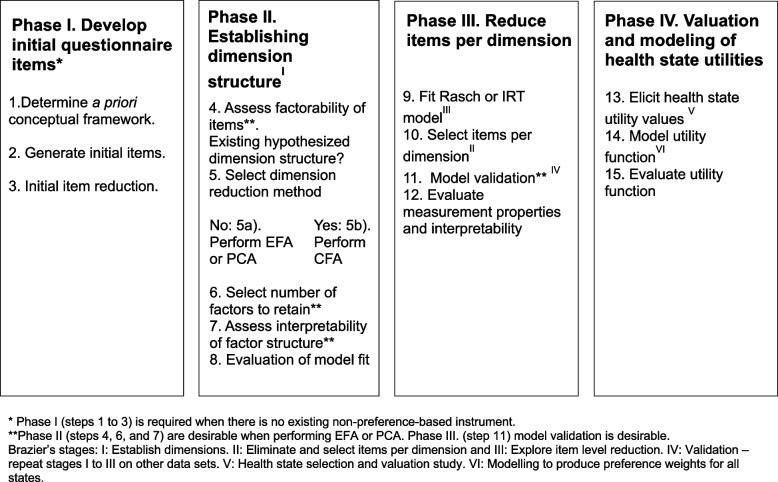
Framework

Figure 2 shows the framework of the four phases and 15 steps of CSPBI development.

#### Phase I (Steps 1–3): Conceptualize measurement construct and develop initial items

These three initial steps were conducted for the 7 instruments developed de novo (Table 2). These steps are only required when developing a CSPBI de novo and therefore are absent from Brazier’s stages, which start with an existing non-preference-based instrument. The data to gather for phase I are the relevant literature of frameworks and existing items, and results from patient interviews or focus groups.

**Table 2:** Phase I (Steps 1–3) Conceptualize measurement construct and develop initial items

	HIV **PB-HIV** [131]	Multiple sclerosis **P-PBMSI** [103–106]	Obesity **WAITe** [108, 109]	Obesity **PBI-WRQL** [111]	Rheumatoid arthritis **MHOM RA** [117]	Vision **Vis-QoL** [118–124]	Diabetes Utility Instrument **DUI** [78]
**Step 1: Determine a priori conceptual framework**							
Conceptual framework			X			X	X
Literature review, existing instruments, chart records							X
Gather expert opinions						P	R, HCP
Analysis method						Grounded theory	
**Step 2: Generate initial items**							
Literature review, existing instruments		X	X		X		
Gather expert opinions			P		HCP	P	
Analysis method			Identify themes based on framework				
**Step 3: Initial item reduction**							
Conceptual framework	X	X		X			
Gather expert opinions	P, HCP		P	P		P	
Analysis method	Removed correlated dimensions; alignment to framework using mapping		Alignment to framework	Removed correlated dimensions; alignment to frame-work using mapping			

*P* Patients, *G* General public, *C* Carers, *HCP* Health care providers, *R* Researchers

##### Step 1. Determine a priori conceptual framework

A conceptual framework defines the construct to be measured. The purpose of starting with a conceptual framework with defined core dimensions is to ensure that measurement of the construct is comprehensive and has established boundaries [132, 133]. Three instruments were developed with condition-specific conceptual frameworks (DUI, WAITe, Vis-QoL) [78, 108, 118]. Frequently, developers reviewed the literature [70, 75, 103, 134] and conducted focus groups to create an a priori framework [70, 91, 118]. Other developers analyzed literature and interviews using content analysis [70], grounded theory [118], or framework analysis [92, 93] (Table 2).

##### Step 2. Generate initial items

The purpose of generating an initial comprehensive pool of items is to cover the entire construct to be measured [20]. Items that represented the conceptual framework of the descriptive system were generated using literature reviews (WAITe)[108], chart reviews, or other existing HRQoL instruments [31, 78, 103, 117, 125, 134, 135]. Patient and clinician experts were consulted in interviews (WAITe) [108], and focus groups (MHOM RA, VisQoL) [42, 117, 118] (Table 2), which consider patient perspectives [136].

##### Step 3. Initial item reduction

The purpose of initial item reduction is to ensure alignment of the items with an a priori framework of HRQoL [103] (Table 2), and to remove redundant items [20]. Developers field-tested the VisQoL in people with and without vision impairment [118, 126]. Developers reduced items after consultation with patients, carers, and/or clinicians (MHOM RA, WAITe, Vis-QoL) [108, 117, 118], performing framework analysis (WAITe) [108]. Development of the PBI-WRQL [111] and PB-HIV [131] removed correlated dimensions (r > / = 0.3) and mapped initial items to an established framework to establish the instrument dimensions (Table 2).

#### Phase II (Steps 4–8): Establishing dimension structure

Factor and principal component analyses (PCA) are data aggregation techniques that explain the pattern of correlations between items and latent constructs, such as HRQoL dimensions [28] (Table 3). Phase II overlaps with Brazier’s stage I (Fig. 2). The intent of establishing the dimensional structure is to assess structural independence, which means there is a low correlation between dimensions [137]. The data to collect for phase II are responses to the questionnaire.

**Table 3:** Phase II (Steps 4 to 8) Establish the dimension structure

	ABC-UI [31, 32]	AQL-5D [33–39]	IUI [40, 41]	OAB-5D [9, 36, 42–44]	EORTC-8D [7, 45–48]	QLU-C10D [49–58]	HAQ-PBM, QLQ-PBM, MSIS-PBM [61]	FACT-8D [62, 63]	CP-6D [66]	DEMQOL-U & -Proxy-U [8, 71–74]	AD-5D [75–77]	CARIES-QC-U [112]	DUI [78, 79]	DHP-3D & 5D [80]	HASMID-8 & 10 [81, 82]	DMD-QoL-8D [134, 138, 139]	
**Step 4. Assess factorability of items**																	
Item-total correlations^E, C, Pa^	X						X					X					
Bartlett test of sphericity^E, Pb^						X			X								
Kaiser-Meyer Olkin measure proportion of variance^E, Pc^						X			X								
Cronbach’s alpha^d^			X												X		
**Step 5. Select dimension extraction method**																	
Principal components analysis (PCA)	X	X	X	X													
Exploratory factor analysis (EFA)									X	X				X			
Confirmatory factor analysis (CFA)								X									
EFA and CFA						X					X		X			X	
**Step 6. If there is no hypothesized dimensional structure, select the factors to retain**																	
Cattell scree test^**E, P**^				X		X											
Amount of variance explained^**E, P**^ – eigen values		X	X	X	X				X					X			
Parallel analysis^**E, P**^						X											
**Step 7. Assess interpretability of dimension structure**																	
Oblimin rotation						X											
Promax rotation									X								
Varimax rotation					X					X							
Varimax and promax rotation	X																
**Step 8. Evaluate model fit**																	
RMSEA^+^						X					X					X	
SRMR^+^																	
CFI^++^						X					X					X	
TLI^++^						X					X						
Factor loadings				X	X	X	X				X		X	X			
Residual correlations						X											
Cross loading	X	X			X	X							X				
	NEWQoL-6D [84–86]	CORE-6D [88–90]	ReQoL-UI [91–94]	MobQoL-7D [95, 140]	MSIS-8D & -P [27, 97–100]	Neuro-QoL derived NQU [101, 102]	P-PBMSI [103–106]	MF-8D [107]	WAITe [108, 109]	POS-E [116]	MHOM RA [117]	Vis-QoL [118–124]	VFQ-UI [125–130]	MacNew-7D [87]	ECOHIS-4D [114]	BUI [64]	CFQ-8D [69]
**Step 4. Assess factorability of items**																	
Item-total correlations^E, C, Pa^									X							X	X
Bartlett test of sphericity^E, Pb^				X						X				X	X		
Kaiser-Meyer Olkin measure proportion of variance^E, Pc^				X						X				X	X		
Cronbach’s alpha^d^				X							X	X			X	X	
**Step 5. Select dimension reduction method**																	
Principal components analysis (PCA)		X								X	X			X			
Exploratory factor analysis (EFA)	X	X		X	X			X				X			X		X
Confirmatory factor analysis (CFA)			X													X	
EFA and CFA									X								
Cattell scree test^**E, P**^		X		X	X			X						X	X		X
**Step 6. If there is no hypothesized dimensional structure, select the factors to retain**																	
Amount of variance explained^**E, P**^ – eigen values			X	X	X									X	X		X
Parallel analysis^**E, P**^									X	X				X	X		
**Step 7. Assess interpretability of dimension structure**																	
Oblimin rotation																	
Promax rotation		X		X							X						
Varimax rotation					X					X					X		
Varimax and promax rotation														X			
**8. Evaluate model fit**																	
RMSEA^+^			X									X				X	
SRMR^+^																X	
CFI^++^			X									X				X	
TLI^++^																X	
Factor loadings				X				X	X	X				X	**X**	X	X
Cross loading																	
Residual correlations																X	

E: exploratory factor analysis, P: principal components analysis, C: confirmatory factor analysis

Factor loading: > 0.3 or > 0.4. + root mean square error of approximation (RMSEA) and standardized root mean squared residual (SRMR) < 0.08 acceptable, < 0.05 good, + + comparative fit index (CFI) and Tucker-Lewis index (TLI) > 0.9 acceptable, > 0.95 good

^a^Inter-item correlations range: 0.3–0.8

^b^Bartlett’s test of sphericity: Chi-sq, *p*-value < 0.01, < 0.05, confirm presence of correlations among items, to indicate whether EFA would be plausible

^c^KMO: > 0.5, or > 0.8, close to 1.0

^d^Cronbach’s alpha: > 0.7 for group use, > 0.8 for individual use

##### Step 4. Assess factorability of items

The factorability of items indicates whether it is feasible to proceed with factor analysis [28]. Coefficients of 0.3 to 0.8 in a correlation matrix [31, 108, 112] or > 0.70 in Cronbach’s alpha [81, 118] are criteria for factorability. If performing PCA or exploratory factor analysis (EFA), developers also assessed the Bartlett test of sphericity, and Kaiser-Meyer Olkin measure of sampling adequacy [31, 66, 87, 108, 116, 141, 142] (Table 3).

##### Step 5. Select dimension extraction method

Next, developers chose a dimension extraction method. The first consideration is whether or not a hypothesized dimension structure exists (Fig. 2). Given an a priori dimensional structure (e.g., a psychometric instrument), authors performed CFA to test the hypothesis and fit covariances or correlations between items and factors (ReQol-UI, FACT-8D) [62, 94] (Table 3). Without an a priori hypothesis of the dimensional structure, most developers performed EFA (CFQ-8D, CP-6D, DEMQOL-U, and DHP-3D and 5D) [66, 69, 71, 80] or PCA (ABC-UI, AQL-5D, IUI, OAB-5D, CORE-6D, POS-E, MHOM RA) [31, 33, 40, 42, 88, 116, 117]. Some authors performed EFA and then CFA (AD-5D, WAITe, QLU-C10D, and DUI) [75, 78, 108, 141], or vice-versa (DMD-QoL) [134]. PCA was used to reduce a set of variables to a smaller set of components [29] (Fig. 2).

##### Step 6. Select the number of factors to retain

If there was no hypothesized dimensional structure, developers had to decide on the number of factors to retain to best represent the underlying structure of the dataset [28]. In PCA and EFA, developers considered the amount of variance that was explained by the eigen values [33, 45, 80, 87] or visualized in a scree plot [69, 87, 88, 107, 141], or they performed parallel analysis to interpret the scree plots more objectively [87, 108, 116, 141] (Table 3).

##### Step 7. Assess interpretability of dimension structure

Within PCA and EFA, developers assessed the factor-loading matrix for interpretability, or meaning [28]. If necessary, developers improved interpretability using: i) promax and oblimin rotation to produce correlated factors (CORE-6D, MHOM RA, QLU-C10D) [49, 88, 117], and ii) varimax rotation to produce uncorrelated factors (EORTC-8D, DEMQOL-U, MSIS-8D, and POS-E) [45, 70, 97, 116]. Developers of the ABC-UI and MacNew-7D used both types of rotation [31, 87]. These methods of rotation help to achieve a structurally simpler matrix than the original factor loading matrix [28] (Table 3).

##### Step 8. Evaluation of model fit

The purpose of evaluating model fit is to assess whether the model needs revision to fit the data. Developers evaluated global model fit using root mean square error of approximation (RMSEA) and standardized root mean square residual (SRMR) (< 0.08 acceptable, < 0.05 good), and comparative fit index (CFI) and Tucker-Lewis index (TLI) (> 0.9 acceptable, > 0.95 good) [49, 64, 75, 94, 118, 134]. Developers evaluated factor loadings (> 0.3 or > 0.4) to ensure the item loaded sufficiently to the factor. In PCA and EFA, developers considered cross-loading differences (< 0.15, or < 0.2) to assign the item to the dimension with the higher loading (ABC-UI, AQL-5D, EORTC-8D, QLU-C10D, DUI) [31, 33, 45, 78, 141]. If model fit was inadequate using any data aggregation approach, developers re-inspected factor loadings and applied residual correlations to improve overall global fit (e.g.,QLU-C10D, BUI) [49, 64]. Developers of the DMD-QoL found poor initial fit using CFA, but fit was improved in a 3-dimensional hierarchical model using EFA [134] (Table 3).

#### Phase III (Steps 9–13): Reducing items per dimension

Together with Phase II, the purpose of reducing items per dimension in Phase III is to create a preference-based instrument that is amenable to valuation [3]. The data required to perform Phase III are responses to the questionnaire, which can be the same set of data used in Phase II.

##### Step 9. Fit Rasch or IRT model

Rasch and IRT models have different purposes, originating from two diverging traditions. Rasch models belong to a model-based tradition since the model is selected first, and the tests are designed to determine if the data fit the model. Proponents of the Rasch model posit that the Rasch model represents the structure of item responses before they can be used for measurement [143]. In the alternative data-based traditions, different models within the IRT family are explored to find the best fitting model for the available data [144].

Thirty-nine of 41 instruments fit the data to a Rasch model. Six instruments used a Rasch rating scale model, nine used the Rasch partial credit model, and 24 used an unspecified polytymous model. Two instruments fit an IRT graded response model (GRM) (ReQoL-UI, NQU) [94, 101] (Table 4).

**Table 4:** Phase III (Steps 9 to 11) Reducing items per dimension

	ABC-UI [31, 32]	AQL-5D [33–39]	IUI [40, 41]	OAB-5D [9, 36, 42–44]	EORTC-8D [7, 45–48]	QLU-C10D [49–58]	BUI [131]	HAQ-PBM, QLQ-PBM, MSIS-PB [61]	FACT-8D [62, 63]	CP-6D [66]	DEMQOL-U & -Proxy-U [8, 71–74]	AD-5D [75–77]	CARIES-QC-U [112]	DUI [78, 79]	DHP-3D & 5D [80]	HASMID-8 & 10 [81, 82]	DMD-QoL [134, 138, 139]	
**Step 9. Select Rasch or IRT model and fit model**																		
Rasch rating scale				X										X			X	
Rasch partial credit		X	X				X			X								
Unspecified polytymous Rasch	X				X	X		X	X		X	X	X		X	X		
Graded response IRT																		
Response level ordering^a^		X	X	X	X	X	X	X	X		X	X	X		X	X		
Meaningfulness of merged levels																		
Item parameters at logit 0	X		X	X	X	X	X	X	X					X				
Global model fit – item-trait interaction^b^		X		X	X	X	X	X		X	X		X					
Person separation index or person separation reliability^c^		X		X	X		X					X		X				
Infit, Outfit							X			X				X			X	
Item fit residuals^d^		X		X		X	X	X	X		X	X	X					
Local dependence						X	X		X									
Differential item functioning	X		X	X	X	X	X	X	X	X		X	X		X	X	X	
Person fit residuals^e^					X		X					X						
Targeting of scale to persons																		
Unidimensionality – principal components analysis of residuals, independent t-statistics			X								X			X				
**Step 10. Select items per dimension**																		
Item coverage across range of construct						X	X		X	X			X				X	
Floor and ceiling effects				X	X	X	X	X			X		X			X	X	
Missing data (%)	X			X	X		X	X					X				X	
Correlation of item to dimension					X												X	
Item importance and impact^f^	HCP					P	P, HCP		P								X	
**Step 11. Model validation**																		
Expert validation										X			X	X				
Another data-set or split half		X		X														
Meaningful clinical rationale (face and content validity^f^					HCP	HCP, P		HCP, R		HCP, R		R	P, G	HCP, P	HCP			
Alignment with parent psychometric instrument^C, E, P^					X	X												
	NEWQoL-6D [84–86]	CORE-6D [88–90]	ReQoL-UI [91–94]	MobQoL-7D [95, 140]	MSIS-8D & -P [27, 97–100]	Neuro-QoL derived NQU [101, 102]	P-PBMSI [103–106]	MF-8D [107]	WAITe [108, 109]	POS-E [116]	MHOM RA [117]	Vis-QoL [118–124]	VFQ-UI [125–130]	MacNew-7D [87]	ECOHIS-4D [114]	PBI-WRQL [111]	PB-HIV [131]	CFQ-R-8 D [69]
**Step 9. Select Rasch or IRT model and fit model**																		
Rasch rating scale							X		X		X							
Rasch partial credit		X		X	X							X						
Unspecified polytymous Rasch	X							X		X			X	X	X	X	X	X
Graded response IRT			X			X												
Response level ordering^a^		X		X	X			X	X	X			X	X	X	X		X
Meaningfulness of merged levels	X	X								X								
Item parameters at logit 0	X				X			X				X						X
Global model fit – item-trait interaction^b^	X	X			X		X	X	X	X	X	X			X	X		X
Person separation index or person separation reliability^c^		X		X	X		X		X	X	X	X	X		X			X
Infit, outfit				X										X	X			
Item fit residuals^d^	X	X			X		X	X	X	X			X	X	X			X
Local dependence								X	X									X
Differential item functioning	X				X			X	X	X			X	X	X	X		X
Person fit residuals^e^					X		X											X
Targeting of scale to persons		X										X						
Unidimensionality – principal components analysis of residuals, independent t-statistics		X		X			X			X								
**Step 10. Select items per dimension**																		
Item coverage across range of construct		X	X#											X		X		X
Absence of floor and ceiling effects		X											X	X	X			X
Percentage missing data													X					X
Correlation of item to dimension						X	X											X
Item importance and impact^e^									PCA									
**Step 11. Model validation**																		
Expert validation														X				
Another data-set or split half	X	X			X					X		X						
Meaningful clinical rationale (face and content validity^e^						P, HCP		HCP					P	P, HCP		X		
Alignment with parent psychometric instrument^C, E, P^					X													

^a^Adjacent response categories were merged if disordered

^b^Item-trait interaction fit statistics, n.s. chi-square, *p*-value > 0.01 after Bonferroni adjustment

^c^Person separation reliability > 0.7 for group use, or > 0.85 for individual use

^d^Exclude items where fit residuals > 2.5 or < -2.5 – and refit Rasch model

^e^Exclude persons where person fit residuals > 2.5 or < -2.5 and refit Rasch model

^f^P: patients, HCP: health care providers, R: researchers. #Fisher information for graded response model

^f^P: patients, HCP: health care providers, R: researchers, G: general public

Aligned with Brazier’s stage III (explore item level reduction) [5] (Fig. 2), CSPBI developers who conducted Rasch analysis first evaluated item response ordering to collapse disordered categories, or removed items with disordered response options, and re-ran the model. Sometimes developers asked experts to review the language of merged categories for clarity and comprehensiveness (face validity) [84, 88, 116] (Table 4).

Developers who used Rasch analysis then evaluated model fit, item fit, and person fit. Global model fit was assessed with an item-trait interaction $\chi$^2^ (non-significant, with Bonferroni correction) and/or person separation index, similar to Cronbach’s alpha or reliability (> 0.7, or > 0.8) [27, 33, 61, 70, 84, 88, 103, 108, 116, 117, 125, 145]. Many developers reported item and then person fit statistics [31, 40, 78]. Mean item fit residuals and mean person fit residuals, measures of divergence between expected and observed responses for item or person responses, ﻿respectively, were evaluated. Residuals > 2.5 or < -2.5 represent poor fit [27, 33, 61, 70, 84, 88, 103, 108, 116, 125]. Additional chi-square statistics were used to investigate observed vs expected responses for items with a severity level near the person’s HRQoL level (infit) or for all items (outfit) [66, 78, 83, 95, 142], where a significant chi-square means an item misfits the model [19] (Table 4).

Next, some developers assessed local dependence and/or differential item functioning (DIF) to explain poor item fit. Local dependence occurs when the response to one item is linked to another item, evaluated by examining a residual correlation matrix [45, 49, 65, 70, 75, 108, 116]. Locally dependent items, such as trouble taking a short walk and trouble taking a long walk, were combined into different levels of one item in the QLU-C10D [49]. DIF, or item bias, is when individuals with known attributes, such as gender or age with the same level of HRQoL respond differently [31, 65, 66, 75, 78, 83, 89, 103, 116, 127, 142]. Developers iteratively removed items with poor fit and the Rasch model was re-fit (Table 4).

Individuals with large person fit residuals (> 2.5 or < -2.5), representing outliers, were also removed, and the Rasch model was re-fit. Some developers evaluated how well the instrument targeted its respondents, with an expected person location of zero and a standard deviation of 1 [88, 127] (Table 4).

Lastly, some developers tested unidimensionality of the instrument by performing PCA of the item residuals after fitting the Rasch model. The associations between item residuals should be random. The developers of the DUI assessed the percentage of variance attributable to the Rasch factor, and the first residual factor to assess unidimensionality [65, 78]. Next, independent t-tests of person score residuals of items that loaded positively (> 0.30) or negatively (< -0.30) were sometimes performed. If the items in the instrument are strictly unidimensional, the percentage of significant tests should be < 5% (POS-E, P-PBMSI, DEMQOL-U, BUI) [65, 70, 103, 116]. This also can be expressed as a confidence interval for a binomial test of proportions for the significant tests (CORE-6D) [88] (Table 4).

Developers of the ReQoL and the NQU scoring system fit the GRM, an IRT model. The model fit of the ReQoL was evaluated with the sum-score-based item fit statistic (S- $\chi$^2^) [145]. The item information function was calculated to identify the score range where each item covered the most information, and the higher the discrimination parameter, the more information it provides. Test information of the total item pool was calculated, and the range where measurement precision > 0.9 was calculated [101, 145] (Table 4).

##### Step 10. Select items per dimension

The purpose of selecting a small number of items per dimension is so that the health states from the eventual preference-based instrument are amenable to valuation [136]. This step overlaps with Brazier’s stage II [5]. Developers used clinimetric and psychometric criteria to select items whether fitting a Rasch or IRT model. If items fit the Rasch model, most developers selected one item per dimension based on Rasch analysis criteria, conventional psychometrics, and item importance. Developers of the DMD-QoL-8D selected two items for each underlying factor [83]. Representative items for the dimension spanned a range of condition severity (AQL-5D, MSIS-8D, DMD-QoL-8D) [27, 33, 83] (Table 4). Developers retained items with a high correlation between the item and its dimension score (AQL-5D, DMD-QoL-8D) [33, 83], that could adequately discriminate (e.g., QLU-C10D and FACT-8D: early vs late stage cancer) [49, 62], or had high responsiveness (e.g., OAB-5D and FACT-8D: standardized response mean between baseline to specific time on treatment) [42, 62]. Conventional psychometric criteria were applied to exclude items with a high proportion of missing data (VFQ-UI, ABC-UI, DMD-QoL-8D) [31, 83, 126], or high floor and ceiling effects (VFQ-UI, CARIES-QC-U, DMD-QoL-8D) [83, 112, 126]. Some developers included item importance and impact ratings from experts to guide item selection (ABC-UI, QLU-C10D) [31, 49], or combinations of patient and health care provider perspectives (Table 4).

For the two instruments that used a graded response IRT model for item selection, developers chose items maximizing coverage of the construct, or selected two items per dimension for their item bank (Neuro-QoL) [101] (Table 4)﻿. Items with high Fisher information contribute to higher measurement precision (ReQoL) [94].

##### Step 11. Model validation

The purpose of model validation is to evaluate whether the fitted model measures what it intended to measure [20]. Aligned with Brazier’s stage IV [5] (Fig. 2), some developers validated the factor analysis or Rasch analysis using another dataset or a split half of the original dataset [27, 33, 43, 78, 84, 88, 116, 118]. Developers incorporated the perspectives of patients, clinicians, or researchers (e.g. importance ratings, interviews) to validate the meaningfulness of the resulting factors [45, 49, 62, 75, 78, 80, 84, 112]. Other developers checked that the resulting classification system had a dimensional structure aligned with the parent psychometric instrument [45, 49, 97] (Table 4).

##### Step 12. Evaluate measurement properties and interpretability

The purpose of assessing measurement properties (reliability, validity, and responsiveness) of a novel instrument before it is used is so that we can be sure that it consistently measures what it is intended to measure, including changes in health [146]. Interpretability is being able to assign qualitative meaning to quantitative scores [146].

Developers evaluated construct validity [41, 63, 67, 72, 95, 103, 117, 127], criterion validity [49, 78, 103, 127] or reliability [34, 49, 63] (Table 5). Responsiveness between baseline and follow-up visits was used to select items (QLU-C10D, EORTC-8D, FACT-8D, AQL-5D) [33, 45, 49, 62]. For example, internal construct validity can be assessed by comparing response distributions with subscales of established instruments (DUI vs SF-12, W-BQ12, and DES) [78]. Criterion or convergent validity was evaluated relative to the parent instrument (IUI vs I-QOL)[40]. Other developers evaluated measurement properties after utilities were elicited (e.g., responsiveness of the DEMQOL-U vs EQ-5D-5L) [8] (Table 5).

**Table 5:** Phase III (Step 12) Evaluate measurement properties and interpretability

	ABC-UI [31, 32]	AQL-5D [33–39]	IUI [40, 41]	OAB-5D [9, 36, 42–44]	EORTC-8D [7, 45–48]	QLU-C10D [49–60]	HAQ-PBM, QLQ-PBM, MSIS-PBM [61]	FACT-8D [62, 63]	CP-6D [66, 67]	DEMQOL-U & -Proxy-U [8, 71–74]	AD-5D [75–77]	CARIES-QC-U [112]	DUI [78, 79]	DHP-3D & 5D [80]	HASMID-8 & 10 [81, 82]	
**Measurement properties**																
**Reliability**																
*Test re-test*						+						+				
Inter-rater																
**Construct validity**																
*Discriminant or known groups*	+	+			+	+		±	+			+	+			
*Convergent*		+	+		+		HAQ-PBM ( ±) QLQ-PBM ( +) MSIS-PBM ( +)	+		+		+				
**Criterion validity**																
*Predictive*			+			+							+			
Responsiveness		+	+	+	+	+	QLQ-PBM ( ±)	+		+		+			+	
**Interpretability**																
Minimal important difference				X						X						
Item wording												X				
	NEWQoL-6D [84–86]	CORE-6D [88–90]	ReQoL-UI [91–94]	MobQoL-7D [95, 140]	MSIS-8D & -P [27, 97–100, 147]	Neuro-QoL derived NQU [101, 102]	P-PBMSI [103–106]	MF-8D [107]	WAITe [108, 109]	POS-E [116]	MHOM RA [117]	Vis-QoL [118–124]	VFQ-UI [125–130]	PB-WRQL [111]	PB-HIV [131]	ECOHIS-4D [148]
**Measurement properties**																
**Reliability**																
*Test re-test*				+			+		+							
*Inter-rater*							+									
**Construct validity**																
*Discriminant or known groups*				+	+	+	+				+	+	+	+	+	+
*Convergent*		+		+	+	+	+		+		+		+		+	
**Criterion validity**																
*Predictive*					+								±			
Responsiveness		±											+	+		+
**Interpretability**																
Minimal important difference																
Item wording															X	

+: Established measurement property, ±: Equivocal measurement property. X: Established

The minimal important difference (MID) was assessed for the OAB-5D and compared with the EQ-5D-5L [9]. Both anchor and distribution-based methods were used to determine the MID of the DEMQOL-U [73] (Table 5).

#### Phase IV (Steps 13–15): Valuation and modeling of health state utilities

Table 6 outlines these steps, which are aligned with Brazier’s stages V and VI [5]. The data required for phase IV are utility weights.

**Table 6:** Phase IV (Step 13–15) Value, model, and evaluate health state utilities

	ABC-UI [31, 32]	AQL-5D [33–39]	IUI [40, 41]	OAB-5D [9, 36, 42–44]	EORTC-8D [7, 45–48]	QLU-C10D [49–58]	HAQ-PBM, QLQ-PBM, MSIS-PBM [61]	FACT-8D [62, 63]	DEMQOL-U & -Proxy-U [8, 71–74]	AD-5D [75–77]	CARIES-QC-U [112, 113]	DUI [78, 79]	DHP-3D & 5D [80]	HASMID-8 & 10 [81, 82]	DMD-QoL-8D [83]	CP-6D [68]	ECHOHIS-4D [115]
**13. Elicit health state utility values**																	
Whose utilities																	
*Patients*										X		X		X			
*General public*	X	X	X	X	X	X	X		X	X	X		X	X	X	X	X
*Carers*									X^+++^	X							
Valuation method																	
*TTO*^*a*^	X	X	X	X	X		X		X				X				
*DCE*^*b*^* or DCE-TTO*^*c*^						X^++^		X		X	X			X	X	X	X
*VAS*^*d*^			X									X					
*EQ-VAS*																	
*BWS*^*e*^										X	X						
*RS*^*f*^																	
*SG*^*g*^												X					
Method of selecting health states																	
*Orthogonal or balanced design*	X	X		X	X	X^+^	X		X	X			X				
*Single and multi-attribute health states to enable modeling*			X									X					
*D-efficiency*										X	X			X		X	X
*C-efficiency*								X							X		
*Corner states*			X									X					
*Rasch vignette*																	
*Own health state*		X^@^															
*Intermediate and anchor states*		X															X
*Naming health state*																	
**14. Model utility values**																	
*Individual level data*	X	X		X	X	X	X		X	X	X		X	X	X	X	X
*Aggregate (mean) data*	X	X	X	X	X	X		X				X	X				
Functional form																	
*Additive function*		X				X	X	X						X		X	X
*Multiplicative function*		X^	X									X					
Model type																	
*Conditional logit*						X^+^		X		X	X			X	X	X	X
*Mixed logit*						X^+^		X								X	
*Multinomial logit*										X							
*Ordinary least squares*	X	X		X	X				X				X				
*Tobit*																	
*Random effects*							X										
*Multiattribute utility function*			X									X					
Estimation method																	
*Maximum likelihood estimation*	X	X			X												
*Expected a posteriori*				X													
**15. Evaluate utility function**																	
Regression model coefficients*	X	X	X	X	X	X	X	X	X	X	X	X	X	X	X	X	X
Consistency of coefficients with descriptive system**	X	X			X	X	X	X	X	X		X	X	X	X	X	X
Fit statistics																	
*RMSE*^*§*^	X								X				X				
*MAE*^*§§*^	X	X	X	X	X		X		X				X				
*AIC*^*§§§*^	X					X^+^		X		X						X	X
*BIC*^*§§§§*^	X					X^+^		X		X						X	X
*R*^*2*^* / Adjusted R*^*2*^		X	X	X					X		X	X		X^^^^	X^^^^		
	WAITe [110]	NEWQoL-6D [84–86]	CORE-6D [88–90]	ReQoL-UI [91–94]	MSIS-8D & -P [27, 97–100]	Neuro-QoL derived NQU [101, 102]	P-PBMSI [103–106]	MF-8D [107]	MHOM RA [117]	Vis-QoL [118–124]	VFQ-UI [125–130]	PBI-WRQL [111]	PB-WRQL [111]	PB-HIV [131]	CFQ-R-8D [69]	MobQoL-7D [96]	
**13. Elicit health state utility values**																	
Whose utilities																	
*Patients*					X	X	X		X	X	X	X	X	X		X	
*General public*	X	X	X	X	X	X		X			X				X	X	
*Carers*																	
*Valuation method*																	
*TTO*^*a*^	X	X	X	X	X			X	X	X	X				X		
*DCE*^*b*^* or DCE-TTO*^*c*^	X																
*VAS*^*d*^												X				X	
*EQ-VAS*													X	X			
*BWS*^*e*^			X														
*RS*^*f*^							X			X							
*SG*^*g*^						X	X										
Method of selecting health states																	
*Orthogonal or balanced design*		X						X									
*Single and multi-attribute health states to enable modeling*						X	X			X							
*D-efficiency*																	
*C-efficiency*																	
*Corner states*						X	X			X							
*Rasch vignette*			X	X													
*Own health state*													X	X		X	
*Intermediate and anchor states*	X															X	
*Naming health state*					X												
**14. Model utility values**																	
*Individual level data*	X	X		X								X			X	X	
*Aggregate (mean) data*		X	X			X	X			X							
Functional form																	
*Additive function*		X	X		X	X			X		X	X			X		
*Multiplicative function*			X			X	X			X							
*Logistic function*													X	X			
*Conditional logit*																	
*Mixed logit*																	
*Multinomial logit*																	
*Ordinary least squares*		X		X				X			X						
*Tobit*															X		
*Random effects*					X										X		
*Multiattribute utility function*										X							
Estimation method																	
*Maximum likelihood estimation*				X				X									
*Expected *a posteriori																	
**15. Evaluate utility function**																	
Regression model coefficients*	X	X	X	X	X	X	X	X	X		X	X	X	X	X	X	
Consistency of coefficients with descriptive system**		X	X	X	X	X	X	X			X	X	X		X		
Fit statistics																	
*RMSE*^*§*^		X	X	X	X						X						
*MAE*^*§§*^		X		X	X			X							X		
*AIC*^*§§§*^				X											X		
*BIC*^*§§§§*^				X											X		
R^2^ / Adjusted R^2^			X		X						X	X					

+ EORTC-derived QLQ-C10 utility weights were elicited in different countries (Australia, UK, Canada, France, Germany, Netherlands, US) using similar methods. + + EORTC-QLU C10D DCE-_TTO_ valuation task included 11 attributes – the 10 dimensions of the QLU C10D and 1 attribute of time. + + + Carers valued the DEMQOL Proxy-U

a: time trade off (TTO), b: discrete choice experiment (DCE), c: discrete choice experiment – time trade off (DCE-TTO), d: visual analogue scale (VAS), e: best worst scaling (BWS), f: rating scale (RS), g: standard gamble (SG)

@Respondents valued their own health state if they had asthma

^*^Regression model coefficient significance. **Consistency of coefficients with descriptive system parameters i.e., worse health should produce lower utilities. §root mean square error (RMSE), §§: mean absolute error (MAE), §§§: Akaike’s information criterion (AIC), §§§§: Bayesian Information Criterion (BIC)

^Linear parametric models were fitted by Yang, and a multiplicative Bayesian models were fitted by Kharroubi.^^Pseudo R^2^

##### Step 13. Elicit heath state utility values

The purpose of eliciting utility values is to develop a set of utility weights to assign to the health states derived from the instrument [149, 150]. Individuals eliciting utility weights were either patients, members of the general public, or carers. Twenty-five CSPBIs elicited utilities from the general public, the most common group, whereas 13 CSPBIs elicited utilities from patients. Patients produced significantly higher utility values than the general public when assessed for the same instrument (e.g., cognition in MS) (MSIS-8D) [98]. Health states must be selected for valuation, and the most common method was an orthogonal design in which each dimension level had an equal chance of combination with all other dimension levels in the instrument (15 instruments) (Table 6). Direct utilities were elicited using cardinal (e.g., TTO, SG), or ordinal (e.g., DCE) methods, most frequently using TTO (21 instruments) (Table 6).

##### Step 14. Model utility function

Statistical models are used to generate a set of utility weights for responses from the CSPBI questionnaire [150]. Utilities of individual [31, 43, 80, 84], aggregate [31, 40, 43, 80, 84, 89], or re-scaled data were modeled [89]. Developers modeled utility values using additive or multiplicative models. Additive models were logit (conditional, mixed, or multinomial), ordinary least squares [31, 43, 80, 83, 84], or random effects models [61, 99]. Multiplicative models fit multiattribute utility functions [40, 79, 119]. Two groups of developers used pseudo preferences by regressing EQ-VAS on each dimension’s response option to create preference weights (PB-WRQL, PB-HIV) [111, 131] (Table 6).

##### Step 15. Evaluate utility function

Developers used various criteria to evaluate the utility function used to score the CSPBI. In our scoping review, the utility function was evaluated based on regression model coefficients for statistical significance [31, 43, 45, 80, 83, 89], and for consistency with the descriptive systems [31, 45, 80, 83]. For example, individuals with poor health were expected to have lower utility values than people with good health. Developers also evaluated the relationship between observed and model-derived utility values [31, 40, 80], or compared model-derived values with EQ-5D utilities [61]. Predictive validity of the algorithm compared estimated and observed utilities using sum of total differences, mean of differences, mean of absolute differences, overall standard deviation of differences, and intraclass correlation coefficient (ICC) [40]. Developers then assessed model fit using root mean square error (RMSE) [31, 80, 84, 89], mean absolute error (MAE) [31, 43, 45, 80, 84], Akaike’s Information Criterion (AIC), and Bayesian Information Criterion (BIC) [31, 49], where smaller values indicate better fit. Model fit also was assessed with adjusted R^2^ which is the proportion of variation explained by the model, where values approaching 1 are better [40, 43, 89, 125] (Table 6).

Figure 2 shows our 15-step framework with Brazier’s corresponding stages.

## Discussion

This scoping review produced a framework with 15 key steps that outline the phases of developing CSPBIs from the development of a conceptual framework to evaluating the utility function. This framework overlaps with the steps or stages from existing frameworks from psychometrics [16], and factor analysis [29, 151], and augments Brazier’s six stages of CSPBI development [5]. Brazier’s stages begin at our step 4 with establishing dimensionality of a pre-existing non-preference-based instrument. We added steps 1–3, required when developing any instrument de novo, coinciding with psychometric item development.

Our framework is novel by connecting the steps of initial stages of psychometric item development (phase I) established by Guyatt et al. (1986)’s seven stages of questionnaire design [16], with the steps of preference-instrument development. Our framework steps, excluding step 1 (a priori framework), are found within Guyatt’s stages [16], but in a different order due to their emphasis on judgemental approaches in creating a psychometric questionnaire vs our focus on quantitative approaches to developing a preference-based questionnaire. We have noted that some steps in Phase II are desirable when performing EFA or PCA, but they are not required. In circumstances where data availability is limited, model validation using a novel dataset may not be possible.

Through comparing our approach with O’Brien [28], and Norman and Streiner [29], our framework generalizes those authors’ approaches that are common amongst factor analysis with and without an a priori factor structure.

Deductive and inductive methods could be combined to reduce initial items. Deductive methods include selecting an a priori framework, using a Rasch or IRT model in item reduction, or hypothesis testing using CFA. Inductive methods include generating items from experts and performing EFA or PCA. Some developers used the results of EFA to inform hypothesis testing on another dataset to fit CFA models [62, 94].

Structural independence, where every health state defined by the set of attributes and levels is possible, could be better emphasized in the development of CSPBI, since it is a defining feature of preference-based instruments [152]. Structural independence can be inferred from factor analysis or evaluated using methods such as Rasch analysis [5], k-means cluster analysis [153], or pairwise independence of attributes [154, 155]. Only one instrument developer explicitly mentioned evaluating structural independence [45].

Surprisingly, few developers used CFA, even though most instruments were developed from existing psychometric instruments, when an a priori dimensional structure could be tested. When evaluating factor loadings, developers did not explicitly state the need to have 2 or 3 items per factor, or that a key objective in EFA is to fit the most parsimonious factor structure [29].

While 39 of 41 instruments used Rasch analysis, fewer than half of CSPBI developers explicitly described using psychometric and Rasch criteria in item selection (step 10), a critical step in this framework.

Peasgood et al. [136] described additional item selection criteria which are being applied in developing the novel generic preference-based instrument, the EQ-HWB (health and well-being) [156]. Some of these criteria overlap with the concept of sensibility [157] and coverage of the full range of the domain in our item selection step. Peasgood et al. also highlight ensuring measurement of current HRQoL so that items can be used in comparisons between and within people, and ensuring that the items are suitable for valuation [136].

The utility elicitation method, respondent type (general public vs patients), and the functional form likely affected the derived utility values but these were frequently not acknowledged and could be further studied [101, 158].

Limitations of this scoping review were not including a critical appraisal of included articles and only included CSPBIs in which Rasch analysis or IRT analysis were used in the steps of their development.

## Conclusions

This study fills a gap in the methodological literature by providing a comprehensive framework to guide the development of preference-based instruments de novo, adding to quality assessment criteria of patient-reported outcomes such as the Consensus-based Standards for the selection of health Measurement Instruments (COSMIN) [146, 159]. Rasch and IRT methods improved item selection and the overall robustness of the resulting instruments with potential for item banking and computerized adaptive testing [101, 158]. This study will help guide the rigorous development of CSPBIs, to better measure patient preferences for clinical decision-making and cost-effectiveness analyses.

### Supplementary Information

**Supplementary Material 1.**

## Data Availability

Data sharing is not applicable to this article as no datasets were generated or analysed during the current study.
