# Supplementary material for: A scoping review to create a framework for the steps in developing condition-specific preference-based instruments de novo or from an existing non-preference-based instrument: use of item response theory or Rasch analysis
Source: Health Qual Life Outcomes. 2024 May 14;22:38. doi: 10.1186/s12955-024-02253-y (PMC11094879; doi:10.1186/s12955-024-02253-y)
Supplement: Supplementary file 1 — Supplementary Material 1. [file 12955_2024_2253_MOESM1_ESM.docx]

## Supplementary Information

S1: Preferred Reporting Items for Systematic reviews and Meta-Analyses extension for Scoping Reviews (PRISMA-ScR) Checklist

S2: Search results from specific databases

S3: Instrument-specific search results from Pubmed

S4: Data extraction form

S5: List of Abbreviations

**S1: Preferred Reporting Items for Systematic reviews and Meta-Analyses extension for Scoping Reviews (PRISMA-ScR) Checklist**

| **SECTION** | **ITEM** | **PRISMA-ScR CHECKLIST ITEM** | **REPORTED ON PAGE #** |
| --- | --- | --- | --- |
| **TITLE** | | | |
| Title | 1 | Identify the report as a scoping review. | p1 |
| **ABSTRACT** | | | |
| Structured summary | 2 | Provide a structured summary that includes (as applicable): background, objectives, eligibility criteria, sources of evidence, charting methods, results, and conclusions that relate to the review questions and objectives. | p3 |
| **INTRODUCTION** | | | |
| Rationale | 3 | Describe the rationale for the review in the context of what is already known. Explain why the review questions/objectives lend themselves to a scoping review approach. | P7, last paragraph of introduction, starts with “The aim…” |
| Objectives | 4 | Provide an explicit statement of the questions and objectives being addressed with reference to their key elements (e.g., population or participants, concepts, and context) or other relevant key elements used to conceptualize the review questions and/or objectives. | P8, top of page |
| **METHODS** | | | |
| Protocol and registration | 5 | Indicate whether a review protocol exists; state if and where it can be accessed (e.g., a Web address); and if available, provide registration information, including the registration number. | P8, bottom of page |
| Eligibility criteria | 6 | Specify characteristics of the sources of evidence used as eligibility criteria (e.g., years considered, language, and publication status), and provide a rationale. | P9 |
| Information sources* | 7 | Describe all information sources in the search (e.g., databases with dates of coverage and contact with authors to identify additional sources), as well as the date the most recent search was executed. | P8,  Table S1 |
| Search | 8 | Present the full electronic search strategy for at least 1 database, including any limits used, such that it could be repeated. | Table S1 |
| Selection of sources of evidence† | 9 | State the process for selecting sources of evidence (i.e., screening and eligibility) included in the scoping review. | P9 |
| Data charting process‡ | 10 | Describe the methods of charting data from the included sources of evidence (e.g., calibrated forms or forms that have been tested by the team before their use, and whether data charting was done independently or in duplicate) and any processes for obtaining and confirming data from investigators. | P9, bottom of page, to top of p10 |
| Data items | 11 | List and define all variables for which data were sought and any assumptions and simplifications made. | Table S2 and Table S3 |
| Critical appraisal of individual sources of evidence§ | 12 | If done, provide a rationale for conducting a critical appraisal of included sources of evidence; describe the methods used and how this information was used in any data synthesis (if appropriate). | Not applicable |
| Synthesis of results | 13 | Describe the methods of handling and summarizing the data that were charted. | P10 |
| **RESULTS** | | | |
| Selection of sources of evidence | 14 | Give numbers of sources of evidence screened, assessed for eligibility, and included in the review, with reasons for exclusions at each stage, ideally using a flow diagram. | p10-11  Figure 1 |
| Characteristics of sources of evidence | 15 | For each source of evidence, present characteristics for which data were charted and provide the citations. | P10-11  Table 1 |
| Critical appraisal within sources of evidence | 16 | If done, present data on critical appraisal of included sources of evidence (see item 12). | Not applicable |
| Results of individual sources of evidence | 17 | For each included source of evidence, present the relevant data that were charted that relate to the review questions and objectives. | Figure 1 |
| Synthesis of results | 18 | Summarize and/or present the charting results as they relate to the review questions and objectives. | P11-21  Tables 1-6 |
| **DISCUSSION** | | | |
| Summary of evidence | 19 | Summarize the main results (including an overview of concepts, themes, and types of evidence available), link to the review questions and objectives, and consider the relevance to key groups. | P21 |
| Limitations | 20 | Discuss the limitations of the scoping review process. | P23 |
| Conclusions | 21 | Provide a general interpretation of the results with respect to the review questions and objectives, as well as potential implications and/or next steps. | P23 |
| **FUNDING** | | | |
| Funding | 22 | Describe sources of funding for the included sources of evidence, as well as sources of funding for the scoping review. Describe the role of the funders of the scoping review. | p27 |

JBI = Joanna Briggs Institute; PRISMA-ScR = Preferred Reporting Items for Systematic reviews and Meta-Analyses extension for Scoping Reviews.

* Where *sources of evidence* (see second footnote) are compiled from, such as bibliographic databases, social media platforms, and Web sites.

† A more inclusive/heterogeneous term used to account for the different types of evidence or data sources (e.g., quantitative and/or qualitative research, expert opinion, and policy documents) that may be eligible in a scoping review as opposed to only studies. This is not to be confused with *information sources* (see first footnote).

‡ The frameworks by Arksey and O’Malley (6) and Levac and colleagues (7) and the JBI guidance (4, 5) refer to the process of data extraction in a scoping review as data charting*.*

§ The process of systematically examining research evidence to assess its validity, results, and relevance before using it to inform a decision. This term is used for items 12 and 19 instead of "risk of bias" (which is more applicable to systematic reviews of interventions) to include and acknowledge the various sources of evidence that may be used in a scoping review (e.g., quantitative and/or qualitative research, expert opinion, and policy document).

*From:* Tricco AC, Lillie E, Zarin W, O'Brien KK, Colquhoun H, Levac D, et al. PRISMA Extension for Scoping Reviews (PRISMAScR): Checklist and Explanation. Ann Intern Med. 2018;169:467–473. [doi: 10.7326/M18-0850](http://annals.org/aim/fullarticle/2700389/prisma-extension-scoping-reviews-prisma-scr-checklist-explanation).

### S2: Search results from specific databases

**MEDLINE SEARCH**

**Performed by:** Teresa Tsui (Teresa.tsui@utoronto.ca)

**Date:** Dec 30, 2022

**Results sent to:** Teresa Tsui <teresa.tsui@utoronto.ca>

**Databases searched:** Ovid MEDLINE: Epub Ahead of Print, In-Process & Other Non-Indexed Citations, Ovid MEDLINE® Daily and Ovid MEDLINE® 1946-Present

**Search Strategy name:** CTT and IRT in preference-based instrument development-MEDLINE-FINAL

| # | Search terms | Jan 9, 2022 | Dec 30, 2022 |
| --- | --- | --- | --- |
| 1 | quality of life/ or quality adjusted life year/ or health status/ or health status indicators/ or ((qualit$ adj2 life) or ((health-related or "health related") adj3 (qualit$ adj2 life)) or hrql or hrqol or "quality-of-life weights" or (("quality adjusted" or quality-adjusted) adj4 (life-year? or "life year?")) or ("healthy year?" adj3 equivalent?) or qaly? or qol or (health adj3 (status or level?)) or (health adj3 (status or risk) adj3 (index$ or indices or indicator? or appraisal?))).mp. | 565022 | 607065 |
| 2 | patient preference/ or (utility$ or valu$ or preference$ or rank$ or (preference adj3 (patient? or base$ or valu$ or rank$ or weight$)) or ((valuation or qaly) adj3 weight$) or "health state$" or "utility measure$" or "valuation function$" or "multi attribute utility theory" or "multi attribute utility classification" or "patient-derived preference$").mp. | 3110841 | 3316915 |
| 3 | ((method$ or procedure? or develop$ or derive$ estimat$ or creat$ or generat$ or construct$ or valua$ or transform$ or validat$ or transfer$ or translat$ or conversi$ or reduc$ or evaluat$).mp. or mt.fs.) adj5 (((focus adj3 group$) or (health adj3 impact?) or interview$ or questionnaire? or respondent? or (survey? adj4 (method$ or baseline or communit$ or round? or health or healthcare or health-care or "health care")) or (patient? adj3 report$ adj3 outcome?) or instrument$ or measure$ or index$ or indices or tool? or scale? or (health adj4 (indicator? or status or risk? or technique?)) or psychometric? or (factor$ adj3 analy$)).mp. or validation studies.pt.) | 1767924 | 1870539 |
| 4 | (focus groups/ or health impact assessment/ or interviews as topic/ or "surveys and questionnaires"/ or exp health care surveys/ or exp health surveys/ or patient reported outcome measures/ or validation studies as topic/ or factor analysis, statistical/ or psychometrics/) adj5 Methods/ | 2298 | 2298 |
| 5 | 3 or 4 | 1769834 | 1872450 |
| 6 | (Rasch or "Item response theory" or "Modern test theory" or "Differential item functioning" or "Unidimention$" or "Latent construct" or "Latent trait theory" or "Local independence" or Mokken or "Maximum likelihood method").mp. | 11748 | 12750 |
| 7 | 1 and 2 and 5 and 6 | 427 | 466 |

**EMBASE SEARCH**

**Performed by:** Teresa Tsui (teresa.tsui@ utoronto.ca)

**Date:** December 30, 2022

**Results sent to:** Teresa Tsui <teresa.tsui@utoronto.ca>

**Databases searched:** **Embase Classic+Embase**1947 to 2022 December 29

**Search Strategy name:** CTT and IRT in preference-based instrument development-EMBASE-FINAL

| **#** | **Searches** | **Jan 9, 2022** | **Dec 30, 2022** |
| --- | --- | --- | --- |
| 1 | "quality of life"/ or quality adjusted life year/ or health status/ or health status indicators/ or "quality of life assessment"/ or ((qualit$ adj2 life) or ((health-related or "health related") adj3 (qualit$ adj2 life)) or hrql or hrqol or "quality-of-life weights" or (("quality adjusted" or quality-adjusted) adj4 (life-year? or "life year?")) or ("healthy year?" adj3 equivalent?) or qaly? or qol or (health adj3 (status or level?)) or (health adj3 (status or risk) adj3 (index$ or indices or indicator? or appraisal?))).mp. | 874000 | 953044 |
| 2 | patient preference/ or (utility$ or valu$ or preference$ or rank$ or (preference adj3 (patient? or base$ or valu$ or rank$ or weight$)) or ((valuation or qaly) adj3 weight$) or "health state$" or "utility measure$" or "valuation function$" or "multi attribute utility theory" or "multi attribute utility classification" or "patient-derived preference$").mp. | 4252910 | 4551525 |
| 3 | (health impact assessment/ or interview/ or questionnaire/ or health care survey/ or exp health survey/ or patient-reported outcome/ or validation study/ or factor analysis/ or psychometry/) adj5 procedures/ | 41061 | 42276 |
| 4 | ((method$ or procedure? or develop$ or derive$ or estimat$ or creat$ or generat$ or construct$ or valua$ or transform$ or validat$ ortransfer$ or translat$ or conversi$ or reduc$ or evaluat$).mp. or mt.fs.) adj5 (((focus adj3 group$) or (health adj3 impact?) or interview$ or questionnaire? or respondent? or (survey? adj4 (method$ or baseline or communit$ or round? or health or healthcare or health-care or "health care")) or (patient? adj3 report$ adj3 outcome?) or instrument$ or measure$ or index$ or indices or tool? or scale? or (health adj4 (indicator? or status or risk or technique?))).mp. or validation studies.pt.) | 1995661 | 2156181 |
| 5 | 3 or 4 | 2015270 | 2176132 |
| 6 | (Rasch or "Item response theory" or "Modern test theory" or "Differential item functioning" or "Unidimention$" or "Latent construct" or "Latent trait theory" or "Local independence" or Mokken or "Maximum likelihood method").mp. | 26255 | 28972 |
| 7 | 1 and 2 and 5 and 6 | 490 | 570 |

**PsycINFO SEARCH**

**Performed by:** Teresa Tsui (Teresa.tsui@ utoronto.ca)

**Date:** December 30, 2022

**Results sent to:** Teresa Tsui <teresa.tsui@utoronto.ca>

**Databases searched:** **PsycINFO**1806 to January Week 1 2022

**Search Strategy name:** CTT and IRT in preference-based instrument development- PsycINFO -FINAL

Search Strategy:

| **#** | **Searches** | Jan 9, 2022 | Dec 30, 2022 |
| --- | --- | --- | --- |
| 1 | "Quality of Life"/ or ((qualit$ adj2 life) or ((health-related or "health related") adj3 (qualit$ adj2 life)) or hrql or hrqol or "quality-of-life weights" or (("quality adjusted" or quality-adjusted) adj4 (life-year? or "life year?")) or ("healthy year?" adj3 equivalent?) or qaly? or qol or (health adj3 (status or level?)) or (health adj3 (status or risk) adj3 (index$ or indices or indicator? or appraisal?))).mp. | 141450 | 130212 |
| 2 | Client Attitudes/ or preferences/ or Preference Measures/ or (utility$ or valu$ or preference$ or rank$ or (preference adj3 (patient? or base$ or valu$ or rank$ or weight$)) or ((valuation or qaly) adj3 weight$) or "health state$" or "utility measure$" or "valuation function$" or "multi attribute utility theory" or "multi attribute utility classification" or "patient-derived preference$").mp. | 625707 | 470854 |
| 3 | (Group Discussion/ or Interviewing/ or surveys/ or questionnaires/ or Self-Report/ or Test Validity/ or factor analysis/ or psychometrics/) adj5 Methodology/ | 2939 | 1756 |
| 4 | (method$ or procedure? or develop$ or derive$ or estimat$ or creat$ or generat$ or construct$ or valua$ or transform$ or validat$ or transfer$ or translat$ or conversi$ or reduc$ or evaluat$).mp. adj5 (((focus adj3 group$) or (health adj3 impact?) or interview$ or questionnaire? or respondent? or (survey? adj4 (method$ or baseline or communit$ or round? or health or healthcare or health-care or "health care")) or (patient? adj3 report$ adj3 outcome?) or instrument$ or measure$ or index$ or indices or tool? or scale? or (health adj4 (indicator? or status or risk? or technique?)) or psychometric? or (factor$ adj3 analy$)).mp. or validation studies.pt.) | 627257 | 526025 |
| 5 | "Item Analysis (Statistical)"/ or "Difficulty Level (Test)"/ or Item Response Theory/ or Classical Test Theory/ or (Rasch or "Item response theory" or "Modern test theory" or "Differential item functioning" or "Unidimention$" or "Latent construct" or "Latent trait theory" or "Local independence" or Mokken or "Maximum likelihood method").mp. | 15988 | 13498 |
| 6 | 3 or 4 | 627809 | 526329 |
| 7 | 1 and 2 and 5 and 6 | 179 | 196 |

**COCHRANE SEARCH**

**Performed by:** Teresa Tsui (teresa.tsui@utoronto.ca)

**Date:** December 30, 2022

**Results sent to:** Teresa Tsui <teresa.tsui@utoronto.ca>

**Databases searched:**

Cochrane Database of Systematic Reviews: last year

Cochrane Central Register of Controlled Trials: last year

Cochrane Methodology Register: last year

| **ID** | **Search** | January 26, 2022 | December 30, 2022 |
| --- | --- | --- | --- |
| #1 | MeSH descriptor: [Quality of Life] this term only | 14163 | 16970 |
| #2 | MeSH descriptor: [Quality-Adjusted Life Years] this term only | 522 | 609 |
| #3 | MeSH descriptor: [Health Status Indicators] this term only | 222 | 239 |
| #4 | (((qualit* near/2 life) or ((health-related or "health related") near/3 (qualit* near/2 life)) or hrql or hrqol or "quality-of-life weights" or (("quality adjusted" or quality-adjusted) near/4 (life-year* or "life year*")) or ("healthy year*" near/3 equivalent*) or qaly* or qol or (health near/3 (status or level*)) or (health near/3 (status or risk) near/3 (index* or indices or indicator* or appraisal*)))) (Word variations have been searched) | 14844 | 17858 |
| #5 | #1 or #2 or #3 or #4 | 15502 | 18567 |
| #6 | MeSH descriptor: [Patient Preference] this term only | 725 | 757 |
| #7 | ((utility* or valu* or preference* or rank* or (preference near/3 (patient* or base* or valu* or rank* or weight*)) or ((valuation or qaly) near/3 weight*) or "health state*" or "utility measure*" or "valuation function*" or "multi attribute utility theory" or "multi attribute utility classification" or "patient-derived preference*")) (Word variations have been searched) | 19404 | 20529 |
| #8 | #6 or #7 | 725 | 757 |
| #9 | MeSH descriptor: [Focus Groups] this term only | 1547 | 1690 |
| #10 | MeSH descriptor: [Health Impact Assessment] this term only | 2504 | 2618 |
| #11 | MeSH descriptor: [Interviews as Topic] this term only | 135 | 130 |
| #12 | MeSH descriptor: [Surveys and Questionnaires] this term only | 862 | 742 |
| #13 | MeSH descriptor: [Health Care Surveys] explode all trees | 624 | 621 |
| #14 | MeSH descriptor: [Patient Reported Outcome Measures] this term only | 1895 | 1791 |
| #15 | MeSH descriptor: [Validation Studies as Topic] this term only | 67 | 36 |
| #16 | MeSH descriptor: [Factor Analysis, Statistical] this term only | 918 | 728 |
| #17 | MeSH descriptor: [Psychometrics] this term only | 68 | 88 |
| #18 | #9 or #10 or #11 or #12 or #13 or #14 or #15 or #16 or #17 | 6277 | 6463 |
| #19 | MeSH descriptor: [Methods] this term only | 62387 | 61624 |
| #20 | #18 and #19 | 4301 | 4250 |
| #21 | (((focus near/3 group*) or (health near/3 impact*) or interview* or questionnaire* or respondent* or (survey* near/4 (method* or baseline or communit* or round* or health or healthcare or health-care or "health care")) or (patient* near/3 report* near/3 outcome*) or instrument* or measure* or index* or indices or tool* or scale* or (health near/4 (indicator* or status or risk* or technique*)) or psychometric* or (factor* near/3 analy*))) (Word variations have been searched) | 124728 | 141280 |
| #22 | ((method* or procedure* or develop* or derive* or estimat* or creat* or generat* or construct* or valua* or transform* or validat* or transfer* or translat* or conversi* or reduc* or evaluat*)) (Word variations have been searched) | 105446 | 111169 |
| #23 | #21 and #22 | 105236 | 111080 |
| #24 | (validation studies):pt (Word variations have been searched) | 564 | 579 |
| #25 | #21 near/5 #22 | 0 | 0 |
| #26 | #18 near/5 #19 | 0 | 0 |
| #27 | #18 or #26 or #25 or #24 | 0 | 0 |
| #28 | ((Rasch or "Item response theory" or "Modern test theory" or "Differential item functioning" or "Unidimention*" or "Latent construct" or "Latent trait theory" or "Local independence" or Mokken or "Maximum likelihood method")) (Word variations have been searched) | 119 | 116 |

**CINAHL SEARCH**

**Performed by:** Teresa Tsui (teresa.tsui@utoronto.ca)

**Date:** December 30, 2022

**Results sent to:** Teresa Tsui <teresa.tsui@utoronto.ca>

**Databases searched:** Interface - EBSCOhost Research Databases; Database - CINAHL

| **#** | **Query** | **January 26, 2022** | **December 30, 2022** |
| --- | --- | --- | --- |
| **S5** | **S1 AND S2 AND S3 AND S4** | 23 | 0 |
| S4 | (MH "Rasch Analysis") OR (MH "Item Analysis") OR TX(Rasch or "Item response theory" or "Modern test theory" or "Differential item functioning" or "Unidimention*" or "Latent construct" or "Latent trait theory" or "Local independence" or Mokken or "Maximum likelihood method") | 508 | 103 |
| S3 | (TX((method* or procedure* or develop* or derive* or estimat* or creat* or generat* or construct* or valua* or transform* or validat* or transfer* or translat* or conversi* or reduc* or evaluat*)) N5 (((focus N3 group*) or (health N3 impact*) or interview* or questionnaire* or respondent* or (survey* N4 (method* or baseline or communit* or round* or health or healthcare or health-care or "health care")) or (patient* N3 report* N3 outcome*) or instrument* or measure* or index* or indices or tool* or scale* or (health N4 (indicator* or status or risk* or technique*)) or psychometric* or (factor* N3 analy*)) ) OR (PT "validation studies") OR ( (MH "Focus Groups") OR (MH "Health Impact Assessment") OR (MH "Interviews+") OR (MH "Surveys") OR (MH "Patient-Reported Outcomes") OR (MH "Validity+") OR (MH "Research Instruments") OR (MH "Instrument Validation") OR (MH "Instrument Construction") OR (MH "Psychometrics")) N5 (MH "Research Methodology")) | 29,024 | 0 |
| S2 | (MH "Patient Satisfaction") OR TX(utility* or valu* or preference* or rank* or (preference N3 (patient* or base* or valu* or rank* or weight*)) or ((valuation or qaly) N3 weight*) or "health state*" or "utility measure*" or "valuation function*" or "multi attribute utility theory" or "multi attribute utility classification" or "patient-derived preference*") | 46,336 | 11,332 |
| S1 | (MH "Quality of Life") OR (MH "Quality-Adjusted Life Years") OR (MH "Health Status") OR (MH "Health Status Indicators") OR TX ((qualit* N2 life) or ((health-related or "health related") N3 (qualit* N2 life)) or hrql or hrqol or "quality-of-life weights" or (("quality adjusted" or quality-adjusted) N4 (life-year* or "life year*")) or ("healthy year*" N3 equivalent*) or qaly* or qol or (health N3 (status or level*)) or (health N3 (status or risk) N3 (index* or indices or indicator* or appraisal*))) | 21,706 | 5,908 |

### S3: Instrument-specific search results from Pubmed

**PUBMED SEARCH**

**Performed by:** Teresa Tsui (Teresa.tsui@utoronto.ca)

**Date:** February 25, 2024

**Results sent to:** Teresa Tsui <teresa.tsui@utoronto.ca>

**Databases searched:** Ovid MEDLINE: Epub Ahead of Print, In-Process & Other Non-Indexed Citations, Ovid MEDLINE® Daily and Ovid MEDLINE® 1946-Present

**Search Strategy name:** Instrument specific Pubmed Search History_2024 02 25

| **Search number** | **Query** | **Search Details** | **Results** |
| --- | --- | --- | --- |
| 1 | ABC-ui[Title/Abstract] | "ABC-ui"[Title/Abstract] | 2 |
| 2 | aql-5d[Title/Abstract] | "aql-5d"[Title/Abstract] | 16 |
| 4 | "incontinence utility index"[Title/Abstract] - Schema: all | "incontinence utility index"[Title/Abstract] | 0 |
| 5 | "incontinence utility index"[Title/Abstract] | "incontinence utility index"[Title/Abstract] | 0 |
| 6 | incontinence utility index[Title/Abstract] | ("incontinance"[All Fields] OR "incontinence"[All Fields] OR "incontinences"[All Fields] OR "incontinency"[All Fields] OR "incontinent"[All Fields] OR "incontinents"[All Fields]) AND "utility index"[Title/Abstract] | 8 |
| 7 | Overactive Bladder Questionnaire- 5 Dimensions[Title/Abstract] - Schema: all | Overactive Bladder Questionnaire- 5 Dimensions[Title/Abstract] | 0 |
| 8 | Overactive Bladder Questionnaire- 5 Dimensions[Title/Abstract] | ("urinary bladder, overactive"[MeSH Terms] OR ("urinary"[All Fields] AND "bladder"[All Fields] AND "overactive"[All Fields]) OR "overactive urinary bladder"[All Fields] OR ("overactive"[All Fields] AND "bladder"[All Fields]) OR "overactive bladder"[All Fields]) AND "questionnaire 5 dimensions"[Title/Abstract] | 0 |
| 9 | oab-5d[Title/Abstract] | "oab-5d"[Title/Abstract] | 8 |
| 10 | European Organisation for Research[Title/Abstract] AND Treatment in Cancer Core Quality of Life Questionnaire – 8 Dimensions[Title/Abstract] - Schema: all | European Organisation for Research[Title/Abstract] AND Treatment in Cancer Core Quality of Life Questionnaire - 8 Dimensions[Title/Abstract] | 0 |
| 11 | European Organisation for Research[Title/Abstract] AND Treatment in Cancer Core Quality of Life Questionnaire – 8 Dimensions[Title/Abstract] | "european organisation for research"[Title/Abstract] AND ((("therapeutics"[MeSH Terms] OR "therapeutics"[All Fields] OR "treatments"[All Fields] OR "therapy"[MeSH Subheading] OR "therapy"[All Fields] OR "treatment"[All Fields] OR "treatment s"[All Fields]) AND ("cancer s"[All Fields] OR "cancerated"[All Fields] OR "canceration"[All Fields] OR "cancerization"[All Fields] OR "cancerized"[All Fields] OR "cancerous"[All Fields] OR "neoplasms"[MeSH Terms] OR "neoplasms"[All Fields] OR "cancer"[All Fields] OR "cancers"[All Fields]) AND "Core"[All Fields] AND ("quality of life"[MeSH Terms] OR ("quality"[All Fields] AND "life"[All Fields]) OR "quality of life"[All Fields]) AND ("questionnair"[All Fields] OR "questionnaire s"[All Fields] OR "surveys and questionnaires"[MeSH Terms] OR ("surveys"[All Fields] AND "questionnaires"[All Fields]) OR "surveys and questionnaires"[All Fields] OR "questionnaire"[All Fields] OR "questionnaires"[All Fields])) AND "8 dimensions"[Title/Abstract]) | 0 |
| 12 | eortc-8d[Title/Abstract] | "eortc-8d"[Title/Abstract] | 12 |
| 13 | QLQ-PBM[Title/Abstract] - Schema: all | QLQ-PBM[Title/Abstract] | 0 |
| 14 | QLQ-PBM[Title/Abstract] | QLQ-PBM[Title/Abstract] | 0 |
| 15 | European Organisation for Research[Title/Abstract] AND Treatment in Cancer Core Quality of Life Questionnaire – Preference-Based Measure[Title/Abstract] | "european organisation for research"[Title/Abstract] AND ((("therapeutics"[MeSH Terms] OR "therapeutics"[All Fields] OR "treatments"[All Fields] OR "therapy"[MeSH Subheading] OR "therapy"[All Fields] OR "treatment"[All Fields] OR "treatment s"[All Fields]) AND ("cancer s"[All Fields] OR "cancerated"[All Fields] OR "canceration"[All Fields] OR "cancerization"[All Fields] OR "cancerized"[All Fields] OR "cancerous"[All Fields] OR "neoplasms"[MeSH Terms] OR "neoplasms"[All Fields] OR "cancer"[All Fields] OR "cancers"[All Fields]) AND "Core"[All Fields] AND ("quality of life"[MeSH Terms] OR ("quality"[All Fields] AND "life"[All Fields]) OR "quality of life"[All Fields]) AND ("questionnair"[All Fields] OR "questionnaire s"[All Fields] OR "surveys and questionnaires"[MeSH Terms] OR ("surveys"[All Fields] AND "questionnaires"[All Fields]) OR "surveys and questionnaires"[All Fields] OR "questionnaire"[All Fields] OR "questionnaires"[All Fields])) AND "preference based measure"[Title/Abstract]) | 1 |
| 16 | Functional Assessment of Cancer Therapy – 8 Dimensions[Title/Abstract] - Schema: all | Functional Assessment of Cancer Therapy - 8 Dimensions[Title/Abstract] | 0 |
| 17 | Functional Assessment of Cancer Therapy – 8 Dimensions[Title/Abstract] | (("functional"[All Fields] OR "functional s"[All Fields] OR "functionalities"[All Fields] OR "functionality"[All Fields] OR "functionalization"[All Fields] OR "functionalizations"[All Fields] OR "functionalize"[All Fields] OR "functionalized"[All Fields] OR "functionalizes"[All Fields] OR "functionalizing"[All Fields] OR "functionally"[All Fields] OR "functionals"[All Fields] OR "functioned"[All Fields] OR "functioning"[All Fields] OR "functionings"[All Fields] OR "functions"[All Fields] OR "physiology"[MeSH Subheading] OR "physiology"[All Fields] OR "function"[All Fields] OR "physiology"[MeSH Terms]) AND ("assess"[All Fields] OR "assessed"[All Fields] OR "assessement"[All Fields] OR "assesses"[All Fields] OR "assessing"[All Fields] OR "assessment"[All Fields] OR "assessment s"[All Fields] OR "assessments"[All Fields]) AND ("cancer ther"[Journal] OR ("cancer"[All Fields] AND "therapy"[All Fields]) OR "cancer therapy"[All Fields])) AND "8 dimensions"[Title/Abstract] | 0 |
| 18 | FACT-8D[Title/Abstract] | "FACT-8D"[Title/Abstract] | 5 |
| 19 | breast utility instrument[Title/Abstract] | ("breast"[MeSH Terms] OR "breast"[All Fields] OR "breasts"[All Fields] OR "breast s"[All Fields]) AND "utility instrument"[Title/Abstract] | 5 |
| 20 | Cerebral Palsy-6 Dimensions[Title/Abstract] - Schema: all | Cerebral Palsy-6 Dimensions[Title/Abstract] | 0 |
| 21 | Cerebral Palsy-6 Dimensions[Title/Abstract] | (("cerebrally"[All Fields] OR "cerebrum"[MeSH Terms] OR "cerebrum"[All Fields] OR "cerebral"[All Fields] OR "brain"[MeSH Terms] OR "brain"[All Fields]) AND "Palsy-6"[All Fields]) AND "Dimensions"[Title/Abstract] | 0 |
| 22 | cp-6d[Title/Abstract] | "cp-6d"[Title/Abstract] | 4 |
| 23 | Dementia Quality of Life-Utility[Title/Abstract] | ("dementia"[MeSH Terms] OR "dementia"[All Fields] OR "dementias"[All Fields] OR "dementia s"[All Fields]) AND "quality of life utility"[Title/Abstract] | 2 |
| 24 | demqol-u[Title/Abstract] | "demqol-u"[Title/Abstract] | 11 |
| 25 | ad-5d[Title/Abstract] | "ad-5d"[Title/Abstract] | 5 |
| 26 | Alzheimer’s Disease-5 Dimensions[Title/Abstract] | (("alzheime s"[All Fields] OR "alzheimer disease"[MeSH Terms] OR ("alzheimer"[All Fields] AND "disease"[All Fields]) OR "alzheimer disease"[All Fields] OR "alzheimer"[All Fields] OR "alzheimers"[All Fields] OR "alzheimer s"[All Fields] OR "alzheimers s"[All Fields]) AND "Disease-5"[All Fields]) AND "Dimensions"[Title/Abstract] | 2 |
| 27 | Diabetes Utility Index[Title/Abstract] | "diabetes utility index"[Title/Abstract] | 2 |
| 28 | dhp-3d[Title/Abstract] | "dhp-3d"[Title/Abstract] | 1 |
| 29 | hasmid[Title/Abstract] | "hasmid"[Title/Abstract] | 4 |
| 30 | Duchenne Muscular Dystrophy Quality of Life-8 Dimensions [Title/Abstract] - Schema: all | Duchenne Muscular Dystrophy Quality of Life-8 Dimensions [Title/Abstract] | 0 |
| 31 | Duchenne Muscular Dystrophy Quality of Life-8 Dimensions [Title/Abstract] | (("muscular dystrophy, duchenne"[MeSH Terms] OR ("muscular"[All Fields] AND "dystrophy"[All Fields] AND "duchenne"[All Fields]) OR "duchenne muscular dystrophy"[All Fields] OR ("duchenne"[All Fields] AND "muscular"[All Fields] AND "dystrophy"[All Fields])) AND ("qualities"[All Fields] OR "quality"[All Fields] OR "quality s"[All Fields])) AND "life 8 dimensions"[Title/Abstract] | 0 |
| 32 | dmd-QoL-8d[Title/Abstract] | "dmd-QoL-8d"[Title/Abstract] | 1 |
| 33 | NEWQOL-6D[Title/Abstract] | "NEWQOL-6D"[Title/Abstract] | 7 |
| 34 | mcnew[Title/Abstract] | "mcnew"[Title/Abstract] | 13 |
| 35 | MacNew Heart Disease Health-Related Quality of Life Instrument -7 Dimensions [Title/Abstract] - Schema: all | MacNew Heart Disease Health-Related Quality of Life Instrument -7 Dimensions [Title/Abstract] | 0 |
| 36 | MacNew Heart Disease Health-Related Quality of Life Instrument -7 Dimensions [Title/Abstract] | ("MacNew"[All Fields] AND ("heart diseases"[MeSH Terms] OR ("heart"[All Fields] AND "diseases"[All Fields]) OR "heart diseases"[All Fields] OR ("heart"[All Fields] AND "disease"[All Fields]) OR "heart disease"[All Fields]) AND ("quality of life"[MeSH Terms] OR ("quality"[All Fields] AND "life"[All Fields]) OR "quality of life"[All Fields] OR ("health"[All Fields] AND "related"[All Fields] AND "quality"[All Fields] AND "life"[All Fields]) OR "health related quality of life"[All Fields]) AND ("instrument"[All Fields] OR "instrument s"[All Fields] OR "instrumentation"[MeSH Subheading] OR "instrumentation"[All Fields] OR "instruments"[All Fields] OR "instrumented"[All Fields] OR "instrumenting"[All Fields]) AND "7"[All Fields]) AND "Dimensions"[Title/Abstract] | 0 |
| 37 | pb-hiv[Title/Abstract] | "pb-hiv"[Title/Abstract] | 1 |
| 38 | Preference-based HIV index[Title/Abstract] | "Preference-based"[All Fields] AND "hiv index"[Title/Abstract] | 1 |
| 39 | Clinical Outcomes in Routine Evaluation-Outcome Measure – 6 Dimensions[Title/Abstract] - Schema: all | Clinical Outcomes in Routine Evaluation-Outcome Measure - 6 Dimensions[Title/Abstract] | 0 |
| 40 | Clinical Outcomes in Routine Evaluation-Outcome Measure – 6 Dimensions[Title/Abstract] | (("ambulatory care facilities"[MeSH Terms] OR ("ambulatory"[All Fields] AND "care"[All Fields] AND "facilities"[All Fields]) OR "ambulatory care facilities"[All Fields] OR "clinic"[All Fields] OR "clinic s"[All Fields] OR "clinical"[All Fields] OR "clinically"[All Fields] OR "clinicals"[All Fields] OR "clinics"[All Fields]) AND ("outcome"[All Fields] OR "outcomes"[All Fields]) AND ("routine"[All Fields] OR "routinely"[All Fields] OR "routines"[All Fields] OR "routinization"[All Fields] OR "routinize"[All Fields] OR "routinized"[All Fields] OR "routinizing"[All Fields]) AND "Evaluation-Outcome"[All Fields] AND ("measurability"[All Fields] OR "measurable"[All Fields] OR "measurably"[All Fields] OR "measure s"[All Fields] OR "measureable"[All Fields] OR "measured"[All Fields] OR "measurement"[All Fields] OR "measurement s"[All Fields] OR "measurements"[All Fields] OR "measurer"[All Fields] OR "measurers"[All Fields] OR "measuring"[All Fields] OR "measurings"[All Fields] OR "measurment"[All Fields] OR "measurments"[All Fields] OR "weights and measures"[MeSH Terms] OR ("weights"[All Fields] AND "measures"[All Fields]) OR "weights and measures"[All Fields] OR "measure"[All Fields] OR "measures"[All Fields])) AND "6 dimensions"[Title/Abstract] | 0 |
| 41 | core-6d[Title/Abstract] | "core-6d"[Title/Abstract] | 11 |
| 42 | reqol-ui[Title/Abstract] | "reqol-ui"[Title/Abstract] | 6 |
| 43 | mobqol-7d[Title/Abstract] | "mobqol-7d"[Title/Abstract] | 2 |
| 44 | MSIS-8D[Title/Abstract] | "MSIS-8D"[Title/Abstract] | 9 |
| 45 | MSIS-PBM[Title/Abstract] | "MSIS-PBM"[Title/Abstract] | 1 |
| 46 | NQU[Title/Abstract] | "NQU"[Title/Abstract] | 5 |
| 47 | P-PBMSI[Title/Abstract] | "P-PBMSI"[Title/Abstract] | 1 |
| 48 | MF-8D[Title/Abstract] - Schema: all | MF-8D[Title/Abstract] | 0 |
| 49 | MF-8D[Title/Abstract] | MF-8D[Title/Abstract] | 0 |
| 50 | Myelofibrosis-8 Dimensions[Title/Abstract] | "Dimensions"[Title/Abstract] | 178,648 |
| 51 | Weight-specific Adolescent Instrument for Economic-Evaluation[Title/Abstract] - Schema: all | Weight-specific Adolescent Instrument for Economic-Evaluation[Title/Abstract] | 0 |
| 52 | Weight-specific Adolescent Instrument for Economic-Evaluation[Title/Abstract] | ("Weight-specific"[All Fields] AND ("adolescences"[All Fields] OR "adolescency"[All Fields] OR "adolescent"[MeSH Terms] OR "adolescent"[All Fields] OR "adolescence"[All Fields] OR "adolescents"[All Fields] OR "adolescent s"[All Fields]) AND ("instrument"[All Fields] OR "instrument s"[All Fields] OR "instrumentation"[MeSH Subheading] OR "instrumentation"[All Fields] OR "instruments"[All Fields] OR "instrumented"[All Fields] OR "instrumenting"[All Fields])) AND "for economic evaluation"[Title/Abstract] | 0 |
| 53 | waite[Title/Abstract] | "waite"[Title/Abstract] | 89 |
| 54 | PBI-WRQL[Title/Abstract] | "PBI-WRQL"[Title/Abstract] | 1 |
| 55 | CARIES-QC-U[Title/Abstract] | "CARIES-QC-U"[Title/Abstract] | 1 |
| 56 | ECOHIS-4D[Title/Abstract] | "ECOHIS-4D"[Title/Abstract] | 3 |
| 57 | POS-E[Title/Abstract] | "POS-E"[Title/Abstract] | 11 |
| 58 | hom ra[Title/Abstract] | "hom"[All Fields] AND "ra"[Title/Abstract] | 18 |
| 59 | MHOM RA[Title/Abstract] - Spellcheck off | "MHOM"[All Fields] AND "RA"[Title/Abstract] | 0 |
| 60 | Multiattribute Health Outcome Measure for Rheumatoid Arthritis[Title/Abstract] - Schema: all | Multiattribute Health Outcome Measure for Rheumatoid Arthritis[Title/Abstract] | 0 |
| 61 | Multiattribute Health Outcome Measure for Rheumatoid Arthritis[Title/Abstract] | ("Multiattribute"[All Fields] AND ("health"[MeSH Terms] OR "health"[All Fields] OR "health s"[All Fields] OR "healthful"[All Fields] OR "healthfulness"[All Fields] OR "healths"[All Fields]) AND ("outcome assessment, health care"[MeSH Terms] OR ("outcome"[All Fields] AND "assessment"[All Fields] AND "health"[All Fields] AND "care"[All Fields]) OR "health care outcome assessment"[All Fields] OR ("outcome"[All Fields] AND "measure"[All Fields]) OR "outcome measure"[All Fields])) AND "for rheumatoid arthritis"[Title/Abstract] | 0 |
| 62 | Health Assessment Questionnaire – Preference-Based Measure[Title/Abstract] | (("health"[MeSH Terms] OR "health"[All Fields] OR "health s"[All Fields] OR "healthful"[All Fields] OR "healthfulness"[All Fields] OR "healths"[All Fields]) AND ("assess"[All Fields] OR "assessed"[All Fields] OR "assessement"[All Fields] OR "assesses"[All Fields] OR "assessing"[All Fields] OR "assessment"[All Fields] OR "assessment s"[All Fields] OR "assessments"[All Fields]) AND ("questionnair"[All Fields] OR "questionnaire s"[All Fields] OR "surveys and questionnaires"[MeSH Terms] OR ("surveys"[All Fields] AND "questionnaires"[All Fields]) OR "surveys and questionnaires"[All Fields] OR "questionnaire"[All Fields] OR "questionnaires"[All Fields])) AND "preference based measure"[Title/Abstract] | 94 |
| 63 | haq-pbm[Title/Abstract] - Schema: all | haq-pbm[Title/Abstract] | 0 |
| 64 | haq-pbm[Title/Abstract] | haq-pbm[Title/Abstract] | 0 |
| 65 | Vision Quality of Life Index[Title/Abstract] | ("vision s"[All Fields] OR "vision, ocular"[MeSH Terms] OR ("vision"[All Fields] AND "ocular"[All Fields]) OR "ocular vision"[All Fields] OR "vision"[All Fields] OR "visions"[All Fields] OR "visioning"[All Fields]) AND "quality of life index"[Title/Abstract] | 23 |
| 66 | Visual Function Questionnaire – Utility Index[Title/Abstract] | "visual function questionnaire utility index"[Title/Abstract] | 10 |
| 67 | ((((((((((((((((((((((((((((((((((((ABC-ui[Title/Abstract]) OR (aql-5d[Title/Abstract])) OR (incontinence utility index[Title/Abstract])) OR (oab-5d[Title/Abstract]))) OR (eortc-8d[Title/Abstract])) OR (European Organisation for Research[Title/Abstract] AND Treatment in Cancer Core Quality of Life Questionnaire – Preference-Based Measure[Title/Abstract])) OR (FACT-8D[Title/Abstract])) OR (breast utility instrument[Title/Abstract])) OR (cp-6d[Title/Abstract])) OR (Dementia Quality of Life-Utility[Title/Abstract])) OR (demqol-u[Title/Abstract])) OR (ad-5d[Title/Abstract])) OR (Alzheimer’s Disease-5 Dimensions[Title/Abstract])) OR (Diabetes Utility Index[Title/Abstract])) OR (dhp-3d[Title/Abstract])) OR (hasmid[Title/Abstract])) OR (dmd-QoL-8d[Title/Abstract])) OR (NEWQOL-6D[Title/Abstract])) OR (mcnew[Title/Abstract])) OR (pb-hiv[Title/Abstract])) OR (Preference-based HIV index[Title/Abstract])) OR (core-6d[Title/Abstract])) OR (reqol-ui[Title/Abstract])OR) OR (mobqol-7d[Title/Abstract])) OR (MSIS-8D[Title/Abstract])OR) OR (NQU[Title/Abstract])OR) OR (P-PBMSI[Title/Abstract])) OR (waite[Title/Abstract])) OR (PBI-WRQL[Title/Abstract])) OR (CARIES-QC-U[Title/Abstract])) OR (ECOHIS-4D[Title/Abstract])) OR (POS-E[Title/Abstract])OR) OR (hom ra[Title/Abstract])) OR (Health Assessment Questionnaire – Preference-Based Measure[Title/Abstract])) OR (Vision Quality of Life Index[Title/Abstract])) OR (Visual Function Questionnaire – Utility Index[Title/Abstract]) | "ABC-ui"[Title/Abstract] OR "aql-5d"[Title/Abstract] OR (("incontinance"[All Fields] OR "incontinence"[All Fields] OR "incontinences"[All Fields] OR "incontinency"[All Fields] OR "incontinent"[All Fields] OR "incontinents"[All Fields]) AND "utility index"[Title/Abstract]) OR "oab-5d"[Title/Abstract] OR "eortc-8d"[Title/Abstract] OR ("european organisation for research"[Title/Abstract] AND ((("therapeutics"[MeSH Terms] OR "therapeutics"[All Fields] OR "treatments"[All Fields] OR "therapy"[MeSH Subheading] OR "therapy"[All Fields] OR "treatment"[All Fields] OR "treatment s"[All Fields]) AND ("cancer s"[All Fields] OR "cancerated"[All Fields] OR "canceration"[All Fields] OR "cancerization"[All Fields] OR "cancerized"[All Fields] OR "cancerous"[All Fields] OR "neoplasms"[MeSH Terms] OR "neoplasms"[All Fields] OR "cancer"[All Fields] OR "cancers"[All Fields]) AND "Core"[All Fields] AND ("quality of life"[MeSH Terms] OR ("Quality"[All Fields] AND "Life"[All Fields]) OR "quality of life"[All Fields]) AND ("questionnair"[All Fields] OR "questionnaire s"[All Fields] OR "surveys and questionnaires"[MeSH Terms] OR ("surveys"[All Fields] AND "questionnaires"[All Fields]) OR "surveys and questionnaires"[All Fields] OR "Questionnaire"[All Fields] OR "questionnaires"[All Fields])) AND "preference based measure"[Title/Abstract])) OR "FACT-8D"[Title/Abstract] OR (("breast"[MeSH Terms] OR "breast"[All Fields] OR "breasts"[All Fields] OR "breast s"[All Fields]) AND "utility instrument"[Title/Abstract]) OR "cp-6d"[Title/Abstract] OR (("dementia"[MeSH Terms] OR "dementia"[All Fields] OR "dementias"[All Fields] OR "dementia s"[All Fields]) AND "quality of life utility"[Title/Abstract]) OR "demqol-u"[Title/Abstract] OR "ad-5d"[Title/Abstract] OR ((("alzheime s"[All Fields] OR "alzheimer disease"[MeSH Terms] OR ("alzheimer"[All Fields] AND "disease"[All Fields]) OR "alzheimer disease"[All Fields] OR "alzheimer"[All Fields] OR "alzheimers"[All Fields] OR "alzheimer s"[All Fields] OR "alzheimers s"[All Fields]) AND "Disease-5"[All Fields]) AND "Dimensions"[Title/Abstract]) OR "diabetes utility index"[Title/Abstract] OR "dhp-3d"[Title/Abstract] OR "hasmid"[Title/Abstract] OR "dmd-QoL-8d"[Title/Abstract] OR "NEWQOL-6D"[Title/Abstract] OR "mcnew"[Title/Abstract] OR "pb-hiv"[Title/Abstract] OR ("preference based"[All Fields] AND "hiv index"[Title/Abstract]) OR "core-6d"[Title/Abstract] OR "reqol-ui"[Title/Abstract] OR "mobqol-7d"[Title/Abstract] OR "MSIS-8D"[Title/Abstract] OR "NQU"[Title/Abstract] OR "P-PBMSI"[Title/Abstract] OR "waite"[Title/Abstract] OR "PBI-WRQL"[Title/Abstract] OR "CARIES-QC-U"[Title/Abstract] OR "ECOHIS-4D"[Title/Abstract] OR "POS-E"[Title/Abstract] OR ("hom"[All Fields] AND "ra"[Title/Abstract]) OR ((("health"[MeSH Terms] OR "health"[All Fields] OR "health s"[All Fields] OR "healthful"[All Fields] OR "healthfulness"[All Fields] OR "healths"[All Fields]) AND ("assess"[All Fields] OR "assessed"[All Fields] OR "assessement"[All Fields] OR "assesses"[All Fields] OR "assessing"[All Fields] OR "assessment"[All Fields] OR "assessment s"[All Fields] OR "assessments"[All Fields]) AND ("questionnair"[All Fields] OR "questionnaire s"[All Fields] OR "surveys and questionnaires"[MeSH Terms] OR ("surveys"[All Fields] AND "questionnaires"[All Fields]) OR "surveys and questionnaires"[All Fields] OR "Questionnaire"[All Fields] OR "questionnaires"[All Fields])) AND "preference based measure"[Title/Abstract]) OR (("vision s"[All Fields] OR "vision, ocular"[MeSH Terms] OR ("vision"[All Fields] AND "ocular"[All Fields]) OR "ocular vision"[All Fields] OR "vision"[All Fields] OR "visions"[All Fields] OR "visioning"[All Fields]) AND "quality of life index"[Title/Abstract]) OR "visual function questionnaire utility index"[Title/Abstract] | 370 |

### S4: Data abstraction form adapted and expanded from Goodwin 2016

| **Identifying information** | | | | | | | | | | | | | | |
| --- | --- | --- | --- | --- | --- | --- | --- | --- | --- | --- | --- | --- | --- | --- |
| **First author:** | |  | | | | | | | | **Year:** | | | |  |
| **Condition:** | |  | | | | | | | | | | | | |
| ***de novo (yes / no)*:** | |  | | | | | | | | | | | | |
| **New preference-based instrument name:** | |  | | | | | | | | | | | | |
| **Citation:** | |  | | | | | | | | | | | | |
| **Developing the initial questionnaire** | | | | | | | | | | | | | | |
| **Determining the *a priori* framework** | | Framework: | | | | Lit review (circle)  Y/N | | | | | Type of expert opinion: | | | |
| **Method of analysis** | |  | | | | | | | | | | | | |
| **Generating initial items** | | Framework: | | | | Lit review (circle)  Y/N | | | | | Type of expert opinion: | | | |
| **Method of analysis** | |  | | | | | | | | | | | | |
| **Item reduction** | | Conceptual framework | | | |  | | | | | Type of expert opinion: | | | |
| **Method of analysis** | |  | | | | | | | | | | | | |
| **Classification system** | | | | | | | | | | | | | | |
| **Original instrument:** | | **Dimensions:** | | **Items:** | | | | | **Levels:** | | | | | |
| **New instrument:** | | **Dimensions:** | | **Items:** | | | | | **Levels:** | | | **States:** | | |
| **Dimensions of new instrument (specify)** | |  | | | | | | | | | | | | |
| **Establishing the dimension structure** | | | | | | | | | | | | | | |
| **Tests to assess factorability (specify)** | |  | | | | | | Cronbach’s alpha: | | | | | | |
| **Dimension reduction method (circle)** | | | | | | | | | | | | | | |
| PCA | | EFA | | | | | | CFA | | | | | | |
| **If no *a priori* hypothesis – criteria to select number of factors to retain** | | | | | | | | | | | | | | |
| **Scree test** | | Y/N | | | | | |  | | | | | | |
| **Eigen values** | | Y/N | | | | | |  | | | | | | |
| **Parallel analysis** | | Y/N | | | | | |  | | | | | | |
| **Other** | |  | | | | | |  | | | | | | |
| **Meaningful rationale – input from groups (circle)** | | Patients | HCP | | | | | Researchers | | | | | General public | |
| **Method to improve interpretability of factor structure** | | | | | | | | | | | | | | |
| **Method of rotation (circle)** | | Oblimin | Promax | | | | | Varimax | | | | | Other | |
| **Evaluate model fit (circle)** | | | | | | | | | | | | | | |
| RMSEA | SRMR | CFI | TLI | | | | | Other: | | | | | | |
| Factor loadings | | Residual correlations | | | | | | Cross loadings | | | | | | |
| **Reducing the number of items per dimension** | | | | | | | | | | | | | | |
| **Dataset used:** | | **Sample size:** | | | | | **Population:** | | | | | | | |
| Rasch model type (circle) | | Rating scale | Partial credit | | | | | Not specified | | | | | | |
| Non-Rasch IRT model (specify) | |  | | | | | | | | | | | | |
| **Evaluate item response levels** | | | | | | | | | | | | | | |
| Response level ordering | | Y/N | | | | | | | | | | | | |
| Meaningfulness of levels | | Y/N | | | | | | | | | | | | |
| **Evaluate model fit, item fit, person fit** | | | | | | | | | | | | | | |
| Item parameters at logit 0 | | Y/N | | | | | | | | | | | | |
| Global model fit – item-trait interaction (chi-square) | | Y/N | | | | | | | | | | | | |
| Person separation reliability | | Y/N | | | | | | | | | | | | |
| Item fit (circle) | | Infit | | | | | | Outfit | | | | | | |
| Item fit residuals | | Y/N | | | | | | Local dependence: Y/N | | | | | | |
| Differential item functioning | | Category (e.g., age) | | | | | | Number of groups (e.g., 2) | | | | | | |
| Person fit residuals | | Y/N | | | | | |  | | | | | | |
| Targeting of scale to persons (specify) | |  | | | | | | | | | | | | |
| Test of unidimensionality (specify) | |  | | | | | | | | | | | | |
| **Selecting items per dimension** | | | | | | | | | | | | | | |
| Item coverage – specify range: | |  | | | | | | | | | | | | |
| Item distribution | | Floor effects | | | | Ceiling effects | | | | | Missing data | | | |
| Item correlation to dimension | | Y/N | | | | | | | | | | | | |
| Item importance | | Patients | | | | | | Health care providers | | | | | | |
| Model validation | | Experts (whom): | | | | | | Another dataset: | | | | | | |
| **Notes:** | |  | | | | | | | | | | | | |
| **Measurement properties** | | | | | | | | | | | | | | |
| **Reliability (type)** | |  | | | | | | | | | | | | |
| **Construct validity (type)** | |  | | | | | | | | | | | | |
| **Criterion validity (type)** | |  | | | | | | | | | | | | |
| **Interpretability** | | | | | | | | | | | | | | |
| **MCID** | |  | | | | | | | | | | | | |
| **Notes** | |  | | | | | | | | | | | | |
| **Valuation methods** | | | | | | | | | | | | | | |
| **States per participant** | | | | | | | | **Country:** | | | | | | |
| **Total number of states valued:** | | | | | **No. per participant:** | | | | | | | | | |
| **Sample size:** | | | | | **No. of participants:** | | | | | | | | | |
| **Participants excluded, and reasons** | |  | | | | | | | | | | | | |
| **Valuation method** | | *e.g. interview* | | **Condition label:** | | | | | | | | | | |
| **Rationale for choice of technique** | |  | | | | | | | | | | | | |
| **States worse than dead** | |  | | | | | | | | | | | | |
| **Upper anchor:** | | | | **Lower anchor:** | | | | | | | | | | |
| **Notes** | |  | | | | | | | | | | | | |
| **Elicit health state utility values** | | | | | | | | | | | | | | |
| **Generating health states (circle)** | | Orthogonal | | | | D-efficiency | | | | | C-efficiency | | | |
|  |  | Rasch vignette | | | | Own health state | | | | | Naming health state | | | |
| **Who’s utilities (circle)** | | Patients | | | | General public | | | | | Other: | | | |
| **Valuation method (circle)** | | TTO | | | | DCE | | | | | DCE-TTO | | | |
|  |  | VAS / RS | | | | BWS | | | | | SG | | | |
| **Model utility values** | | | | | | | | | | | | | | |
| **Type of data (circle)** | | Individual | | | | | | Aggregate | | | | | | |
| **Functional form (circle / specify)** | | Additive | | | | Multiplicative | | | | | Other: | | | |
| **Model type (circle)** | | Conditional logit | | | | Mixed logit | | | | | Multinomial logit | | | |
|  |  | Ordinary least squares | | | | Other: | | | | |  | | | |
| **Estimation method** | | ML | | | | EAP | | | | | Other: | | | |
| **Evaluate utility function** | | | | | | | | | | | | | | |
| **Regression model** | |  | | | | | | | | | | | | |
| **Consistency with descriptive system** | |  | | | | | | | | | | | | |
| **Fit statistics (circle / specify)** | | RMSE | | | | MAE | | | | | AIC | | | |
|  |  | BIC | | | | Other: | | | | | | | | |
| **Notes** | |  | | | | | | | | | | | | |

### S5. List of Abbreviations

| **Abbreviation** | **Description** |
| --- | --- |
| ABC-UI | Aberrant Behavior Checklist -Utility Index |
| AD-5D | Alzheimer’s Disease-5 Dimensions |
| AIC | Akaiki's information criterion |
| AQL-5D | Asthma Quality of Life Questionnaire- 5 Dimensions |
| BIC | Bayesian information criterion |
| BUI | Breast Utility Instrument |
| BWS | Best worst scaling |
| C | Carers |
| CARIES-QC-U | Caries Impacts and Experiences Questionnaire for Children Utility version |
| CFA | Confirmatory factor analysis |
| CFI | Comparative fit index |
| CORE-6D | Clinical Outcomes in Routine Evaluation-Outcome Measure – 6 Dimensions |
| CP-6D | Cerebral Palsy-6 Dimensions |
| CFQ-R-8 D | Cystic Fibrosis Questionnaire-Revised-8 Dimensions |
| DCE-TTO | Discrete-choice experiment, time-trade-off |
| DEMQOL-U  DEMQOL-Proxy-U | Dementia Quality of Life-Utility (patient self-report and carer proxy-report) |
| DHP-3D  DHP-5D | Diabetes Health Profile-3 Dimensions and 5-Dimensions |
| DMD-QoL-8D | Duchenne Muscular Dystrophy Quality of Life-8 Dimensions |
| DUI | Diabetes Utility Index |
| ECOHIS-4D | Early Childhood Oral Health Impact Scale-4 Dimensions |
| EFA | Exploratory factor analysis |
| EORTC-8D | European Organisation for Research and Treatment in Cancer Core Quality of Life Questionnaire – 8 Dimensions |
| FACT-8D | Functional Assessment of Cancer Therapy – 8 Dimensions |
| G | General public |
| HAQ-PBM | Health Assessment Questionnaire – Preference-Based Measure |
| HASMID-8 HASMID-10 | Health and Self-Management in Diabetes Questionnaire |
| HCP | Health care providers |
| IRT | Item response theory |
| IUI | Incontinence Utility Index |
| KMO | Kaiser-Meyer Olkin measure proportion of variance |
| LT-TTO | Lead time-time trade-off |
| MacNew | MacNew Heart Disease Health-Related Quality of Life Instrument -7 Dimensions |
| MF-8D | Myelofibrosis-8 Dimensions |
| MHOM RA | Multiattribute Health Outcome Measure for Rheumatoid Arthritis |
| MobQoL-7D | Mobility Quality of Life-7 Dimensions |
| MSIS-8D  MSIS-8D-P | Multiple Sclerosis Impact Scale – 8 Dimensions, and -8 Dimensions patient versions |
| MSIS-PBM | Multiple Sclerosis Impact Scale – Preference-Based Measure |
| N/A | Not applicable |
| NEWQOL-6D | Epilepsy-specific Quality of Life – 6 Dimensions |
| NQU | Health-related quality of life in people with neurological conditions, NeuroQol-Utility System |
| OAB-5D | Overactive Bladder Questionnaire- 5 Dimensions |
| P | Patients |
| P-PBMSI | Preference-Based Multiple Sclerosis Index |
| PB-HIV | Preference-based HIV index |
| PBI-WRQL | Preference-Based Index of Weight-Related Quality of Life |
| PCA | Principal components analysis |
| POS-E | Palliative Care Outcome Scale Descriptive System |
| QLQ-PBM | European Organisation for Research and Treatment in Cancer Core Quality of Life Questionnaire – Preference-Based Measure |
| QLU-C10D | European Organisation for Research and Treatment in Cancer Core Quality of Life Questionnaire – 10 Dimensions |
| R | Researchers |
| ReQoL-UI | Recovering Quality of Life Utility Index |
| RMSEA | Root mean square error of approximation |
| RS | Rating scale |
| SG | Standard gamble |
| SRMR | Standardized root mean squared residual |
| TLI | Tucker-Lewis Index |
| TTO | Time trade-off |
| UK | United Kingdom |
| USA | United States of America |
| VAS | Visual analogue scale |
| VFQ-UI | Visual Function Questionnaire – Utility Index |
| Vis-QoL | Vision Quality of Life Index |
| WAITe | Weight-specific Adolescent Instrument for Economic-Evaluation |
